# Involvement of Auxin-Mediated CqEXPA50 Contributes to Salt Tolerance in Quinoa (*Chenopodium quinoa*) by Interaction with Auxin Pathway Genes

**DOI:** 10.3390/ijms23158480

**Published:** 2022-07-30

**Authors:** Wenjun Sun, Min Yao, Zhen Wang, Ying Chen, Junyi Zhan, Jun Yan, Shuangqing Jiang, Shanshan Jian, Hui Chen, Tongliang Bu, Zizong Tang, Qingfeng Li, Haixia Zhao, Qi Wu

**Affiliations:** 1 College of Life Science, Sichuan Agricultural University, Ya’an 625014, China; sunnan82475@163.com (W.S.); ym2678874153@163.com (M.Y.); wangz761226086@163.com (Z.W.); cy15680687623@163.com (Y.C.); jiangsq1001@163.com (S.J.); jianshanshan0801@163.com (S.J.); tlbu@163.com (T.B.); 3530279123456789@163.com (Z.T.); qingfeng.li@sicau.edu.cn (Q.L.); zhaohaixia@sicau.edu.cn (H.Z.); wuqi@sicau.edu.cn (Q.W.); 2 College of Life Science, Nanjing Agricultural University, Nanjing 210032, China; zhanjunyi0412@163.com; 3 Key Laboratory of Coarse Cereal Processing, Ministry of Agriculture and Rural Affairs, School of Food and Biological Engineering, Chengdu University, Chengdu 610106, China; yanjun6622@gmail.com

**Keywords:** auxin, salt stress, *Cqexpansin*, auxin pathway gene, antioxidant capacity

## Abstract

Soil salinization is a global problem that limits crop yields and threatens agricultural development. Auxin-induced expansins contribute to plant salt tolerance through cell wall loosening. However, how auxins and expansins contribute to the adaptation of the halophyte quinoa (*Chenopodium quinoa*) to salt stress has not yet been reported. Here, auxin was found to contribute to the salt tolerance of quinoa by promoting the accumulation of photosynthetic pigments under salt stress, maintaining enzymatic and nonenzymatic antioxidant systems and scavenging excess reactive oxygen species (ROS). The *Chenopodium quinoa* expansin (Cqexpansin) family and the auxin pathway gene family (*Chenopodium quinoa* auxin response factor (CqARF), *Chenopodium quinoa* auxin/indoleacetic acid (CqAux/IAA), *Chenopodium quinoa* Gretchen Hagen 3 (CqGH3) and *Chenopodium quinoa* small auxin upregulated RNA (CqSAUR)) were identified from the quinoa genome. Combined expression profiling identified *Chenopodium quinoa* α-expansin 50 (*CqEXPA50*) as being involved in auxin-mediated salt tolerance. *CqEXPA50* enhanced salt tolerance in quinoa seedlings was revealed by transient overexpression and physiological and biochemical analyses. Furthermore, the auxin pathway and salt stress-related genes regulated by *CqEXPA50* were identified. The interaction of CqEXPA50 with these proteins was demonstrated by bimolecular fluorescence complementation (BIFC). The proteins that interact with CqEXPA50 were also found to improve salt tolerance. In conclusion, this study identified some genes potentially involved in the salt tolerance regulatory network of quinoa, providing new insights into salt tolerance.

## 1. Introduction

Soil salinization has a serious negative impact on crop development and yield [1], which are increasingly serious global problems [2]. Climate change and improper irrigation strategies have undoubtedly exacerbated soil salinization [3,4]. Salt stress affects plant life by inhibiting germination and regulating development. Salt stress can cause plant water shortages, ion imbalances and ion toxicity [5]. To survive, plants have evolved adaptive mechanisms, including hormonal regulation, stress sensing and gene regulation.

Auxin is reported to play a crucial role in plant tolerance to salt stress [6]. As a driver of plant development, auxin plays an integral role in multiple developmental processes, including flowering [7], root development [8], leaf senescence [9] and leaf morphogenesis [10]. Indole-3-acetic acid (IAA) greatly alleviated the adverse effects of salt stress on maize (*Zea mays* L.) growth and development [11]. Exogenous application of IAA can improve the developmental status, protein content and antioxidant enzyme activity of potato (*Solanum tuberosum* L.) under salt stress [12]. Another study indicated that exogenous spraying of IAA can increase the starch content, yield and filled-grain percentage of rice (*Oryza sativa* L.) grains under salt stress [13].

The cell wall can provide mechanical support for plant cells, as well as the plasticity required to prevent the invasion of external adverse factors [14]. As key growth regulators, expansin proteins play central roles in the control of cell wall plasticity and can continuously assemble, reshape and decompose the cell wall [15]. Expansins are involved in multiple processes, such as root elongation [16], leaf growth [17] and fruit softening [18], by regulating cell wall extension. Some reports suggest that auxin can induce *expansin* gene expression. For example, IAA can improve maize (*Zea mays* L.) leaf development and the expression of the *expansin* gene under salt stress [19]. Overexpression of the rose *RhEXPA4* gene in *Arabidopsis thaliana* promotes salt tolerance by modifying cell expansion and improving plant development [20]. *Nicotiana tabacum* α-expansin 11 (*NtEXPA11*)-overexpressing plants can adapt to stress and have a strong tolerance to salt and drought stress [21]. The rice *OsEXPA7* gene can promote salt tolerance by regulating cell wall loosening, scavenging reactive oxygen species (ROS) and coordinating sodium transport [22]. Although expansins play central roles in both plant development and stress tolerance, there are very limited reports on them in quinoa (*Chenopodium quinoa*).

Quinoa (*Chenopodium quinoa*) is a highly nutritious grain containing a variety of essential amino acids and an extremely high protein content [23]. It is used as a golden crop that can improve world food security [24]. As a halophyte, it can withstand a variety of abiotic stresses [25]. Current research on the salt tolerance of quinoa mainly focuses on agronomic traits [26], and reports on gene regulation are very limited. Identifying salt tolerance-responsive genes and dissecting regulatory networks are crucial to assist plants in coping with salt stress.

This study explored how IAA alleviated the damage of salt stress on quinoa seedlings from a physiological and biochemical perspective, and identified salt tolerance-responsive genes. The functions of these genes in salt tolerance were explored through transient overexpression and physiological, biochemical and molecular biological analyses. This study expands the salt tolerance gene pool and provides a foundation for the breeding of elite varieties.

## 2. Results

### 2.1. Exogenous Auxin Relieves Salt-Induced Growth Inhibition of Quinoa Seedlings

A certain concentration of auxin alleviated the inhibition of quinoa root growth by salt stress (Figure S1A). However, when the IAA concentration was increased to 5 μM, the mitigating effect began to decline (Figure S1A). When the IAA concentration continued to increase, root length (Figure S1B) and fresh weight (Figure S1C) were not significantly different from those under salt stress.

The auxin inhibitor N-1-naphthylphthalamic acid (NPA) was added to determine whether this relieving effect was caused by IAA. When 5 μM NPA was added, the alleviating effect of IAA on the growth of quinoa seedlings under salt stress was already inhibited (Figure S2A). When the NPA concentration reached 7 μM it completely counteracted the effect of IAA, and as the NPA concentration continued to increase there were no additional significant changes in root length (Figure S2B) or fresh weight (Figure S2C). Therefore, we chose 3 μM IAA and 7 μM NPA for subsequent experiments.

**Figure 1:**
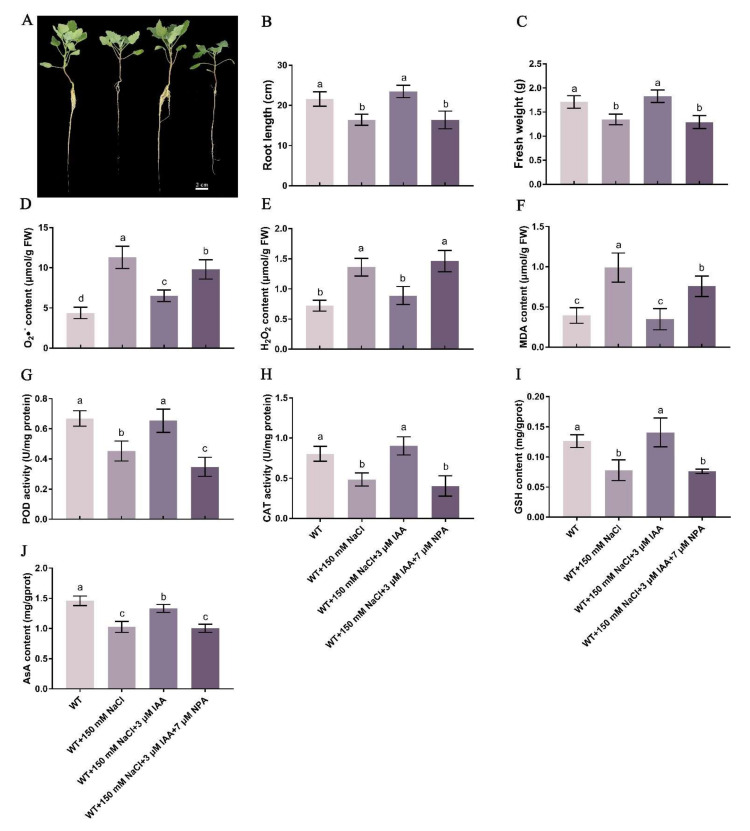
Effect of IAA on the growth and antioxidant capacity of quinoa roots under salt stress. Quinoa seedlings of six true leaves were cultured in Hoagland solution (CK), 150 mM NaCl-Hoagland solution, 150 mM NaCl + 3 μM IAA-Hoagland solution and 150 mM NaCl + 3 μM IAA + 7 μM NPA-Hoagland solution for two weeks and then phenotype, root length and fresh weight were recorded. (**A**) Phenotypic changes in quinoa seedlings under different treatments. Bar = 2 cm; (**B**) The root length; (**C**) The fresh weight. (**D**) O2•^−^ content in roots; (**E**) H_2_O_2_ content in roots; (**F**) MDA content in roots. (**G**) POD activity in roots; (**H**) CAT activity in roots. (**I**) GSH content in roots; (**J**) ASA content in roots. Values are mean ± SD (*n* = 3). Different letters (a–d) in Figure 1B−J indicate significant differences at *p* < 0.05 according to one-way ANOVA (comparing the mean of each column with the mean of every other column) in GraphPad Prism 7.04.

Compared with the control, salt stress inhibited the development of quinoa seedlings, and the root length and fresh weight decreased significantly by 23.9% and 21.2%, respectively (Figure 1A–C). Compared with salt stress alone, the root length and fresh weight of quinoa seedlings were significantly increased by 43% and 35.7% with the addition of IAA (Figure 1B,C). However, the alleviating effect of IAA on salt stress was counteracted by 7 μM NPA (Figure 1A–C).

### 2.2. Effects of IAA on the Contents of Photosynthetic Pigments in Quinoa Seedlings under Salt Stress

Compared with the control, the chlorophyll and carotenoid contents in quinoa leaves under salt stress decreased significantly by 29.9% and 32.9%, respectively (Figure S3A,B). Compared with salt stress alone, the contents of chlorophyll and carotenoids in quinoa leaves increased significantly by 87.9% and 39.2% after IAA addition, respectively (Figure S3A,B). After adding NPA, the content of photosynthetic pigments was not significantly different from that under salt stress, since it counteracts the promoting effect of IAA on photosynthetic pigments under salt stress (Figure S3A,B).

### 2.3. Effects of IAA on the Contents of Superoxide Radical (O_2_•^−^), Hydrogen Peroxide (H_2_O_2_) and Malondialdehyde (MDA) in Quinoa Seedlings under Salt Stress

Compared with the control, the contents of O_2_•^−^, H_2_O_2_ and MDA in quinoa roots under salt stress increased significantly by 158%, 88.6% and 151.8%, respectively (Figure 1D–F). When IAA was added, the content of these indicators decreased significantly by 42.3%, 34.7% and 65%, respectively (Figure 1D–F). However, the addition of NPA plus IAA resulted in the recovery of the contents of these indicators to the levels under salt stress. The contents of O_2_•^−^, H_2_O_2_ and MDA in quinoa shoots were detected and found to be similar to those in roots (Figure S3C–E).

### 2.4. Effects of IAA on Enzyme Activity from Quinoa Seedlings under Salt Stress

The activities of peroxidase (POD) and catalase (CAT) in quinoa roots under salt stress were significantly decreased by 32.3% and 39.7%, respectively, compared with the control (Figure 1G,H). However, when IAA was added, the POD and CAT activities in quinoa roots under salt stress increased significantly by 44.4% and 86.3%, respectively (Figure 1G,H). The addition of NPA counteracted the effect of IAA on POD and CAT activity under salt stress (Figure 1G,H). The trend of POD activity in shoots was the same as that in roots (Figure S3F), whereas the activity of CAT in shoots was completely opposite to that in roots (Figure S3G).

### 2.5. Effects of IAA on Glutathione (GSH) and Ascorbic Acid (ASA) Contents of Quinoa Seedlings under Salt Stress

The contents of GSH and ASA in roots under salt stress were significantly decreased by 38.2% and 29.6%, respectively, compared with the control (Figure 1I,J). Compared with salt stress alone, the GSH and ASA contents in quinoa roots increased significantly by 80.6% and 29.7%, respectively, after adding IAA (Figure 1I,J). However, the contents of GSH and ASA in quinoa roots after adding NPA were not significantly different from those under salt stress alone (Figure 1I,J). The GSH and ASA contents in shoots (Figure S3H,I) were similar to the results in roots (Figure 1I,J).

### 2.6. Comprehensive Analysis of the Cqexpansin Family

**Figure 2:**
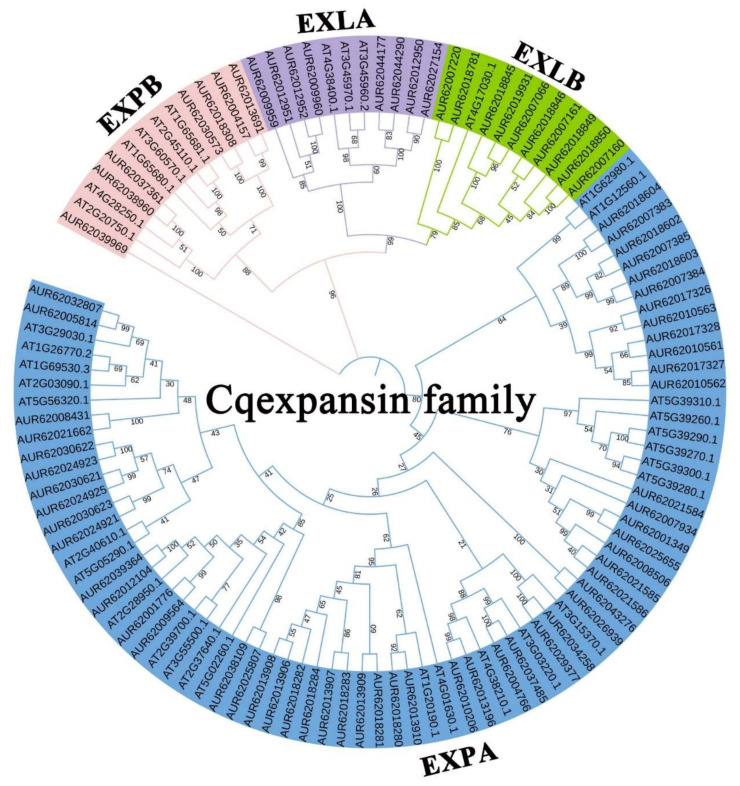
Analysis and identification of Cqexpansin family in quinoa.

To identify *Cqexpansin* genes potentially involved in salt stress in response to IAA, we identified 78 *Cqexpansin* genes from the quinoa genome (Table S1). A maximum likelihood (ML) tree of 78 *Cqexpansin* and 35 *Atexpansin* genes was constructed using the Wheeler and Goldman (WAG) model. These *expansins* were divided into four subfamilies, among which EXPA subfamily contained the most members (Figure 2).

The 78 *Cqexpansins* were also divided into four subgroups (Figure S4A-i). Motif detection revealed that most Cqexpansins contained motif 1, only EXPA subgroup members lacked motifs 9 and 10 and only EXPA subgroup members contained motif 7 (Figure S4A-ii, Table S6). Gene structure analysis showed that all *Cqexpansins* contained conserved domains with introns ranging from 1 to 21, with members of the EXLA subfamily having the highest number of introns (Figure S4A-iii). A total of 22 tandem duplicated and 17 segmentally duplicated *Cqexpansin* genes were detected (Figure S4B,C, Table S11).

Inferring the syntenic relationship between *Cqexpansins* and *expansins* in representative plants, it was found that the number of syntenic gene pairs was 43 in soybean (*Glycine max*), 24 in beet (*Beta vulgaris*), 23 in tomato (*Solanum lycopersicum*), 14 in tartary buckwheat (*Fagopyrum tataricum*), 13 in rice (*Oryza sativa*) and 5 in *Arabidopsis thaliana* (Figure S4D, Table S16).

Phylogenetic tree construction and subfamily classification of expansin family in quinoa and *Arabidopsis thaliana*. Maximum likelihood tree based on the full-length sequences of the 35 *Arabidopsis thaliana expansin* genes and 87 quinoa *expansin* genes were constructed under WAG model using Mega 7.

### 2.7. Exogenous IAA-Mediated Expression of Cqexpansins in Quinoa Seedlings under Salt Stress

**Figure 3:**
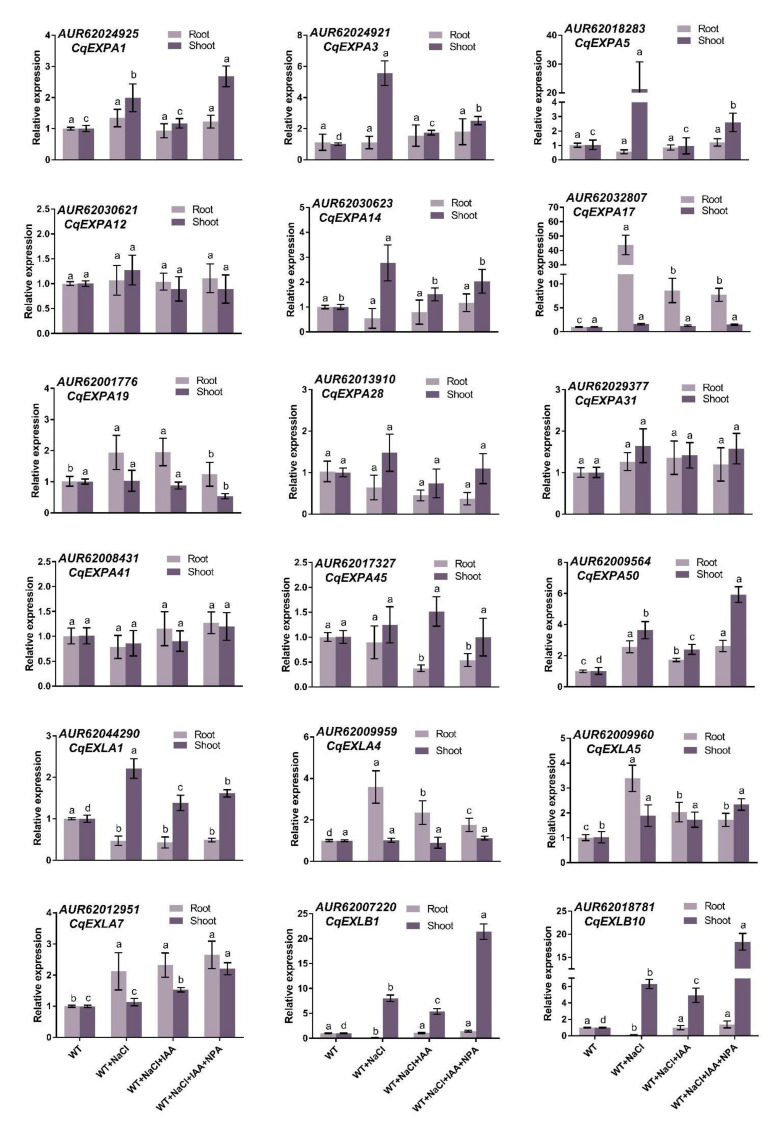
Effects of IAA and NPA on the expressions of *Cqexpansin* genes in quinoa root and shoot under salt stress. Quinoa seedlings of six true leaves were cultured in Hoagland solution (CK), 150 mM NaCl-Hoagland solution, 150 mM NaCl + 3 μM IAA-Hoagland solution and 150 mM NaCl + 3 μM IAA + 7 μM NPA-Hoagland solution for two weeks, and then qRT-PCR was used to detect the expression of different genes. Values are mean ± SD (*n* = 3). Different letters (a–d) in Figure 3 indicate significant differences at *p* < 0.05 according to one-way ANOVA (comparing the mean of each column with the mean of every other column) in GraphPad Prism 7.04.

Expression analysis was performed to identify *Cqexpansins* in response to IAA-mediated salt stress. The expression patterns of *Cqexpansins* vary widely. Under salt stress, the expression levels of five *Chenopodium quinoa* α-expansin (*CqEXPA*) genes (*CqEXPA1*, *CqEXPA3*, *CqEXPA5*, *CqEXPA14* and *CqEXPA50*) were significantly increased in shoots, while the expression levels of three *CqEXPA* genes (*CqEXPA17*, *CqEXPA19* and *CqEXPA50*) were significantly increased in roots (Figure 3). Under salt stress, the expression of the *Chenopodium quinoa* expansin-like A 1 (*CqEXLA1*) gene was induced in shoots, while the expression levels of three *CqEXLA* (*CqEXLA4*, *CqEXLA5* and *CqEXLA7*) genes were significantly increased in roots (Figure 3). Under salt stress, the expression levels of the two *Chenopodium quinoa* expansin-like B (*CqEXLB*) genes (*CqEXLB1* and *CqEXLB10*) increased significantly in shoots but decreased significantly in roots (Figure 3). It is worth noting that the expression levels of *CqEXPA50* in roots and shoots under salt stress were 2.6- and 3.6-fold that of the control group (Figure 3). The expression levels of *CqEXPA50* in roots and shoots decreased after adding IAA, while the addition of NPA on the basis of IAA restored its high expression levels (Figure 3). Therefore, *CqEXPA50* was selected for further exploration.

### 2.8. Subcellular Localization of CqEXPA50

The pCAMBIA1300-CqEXPA50 recombinant plasmid was transiently transformed into tobacco leaves. By observing the fluorescent signal, it was confirmed that CqEXPA50 was localized to the nucleus and cytoplasm (Figure S5).

### 2.9. Phenotype and Leaf Photosynthetic Pigment Contents of Quinoa Transiently Overexpressing CqEXPA50

**Figure 4:**
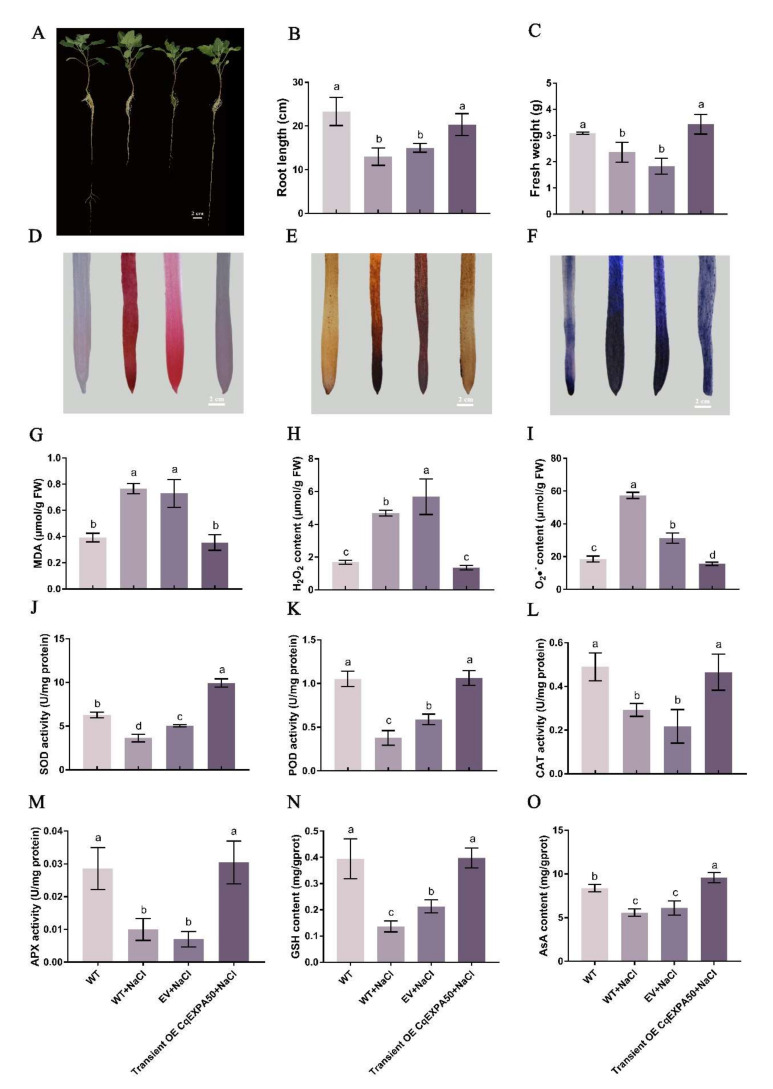
Effects of CqEXPA50 on the growth and antioxidant capacity of quinoa roots under salt stress. The phenotype, root length and fresh weight of all these quinoa seedlings in different treatments were recorded. (**A**) Phenotypic changes in quinoa seedlings under different treatments. Bar = 2 cm. (**B**) The root length. (**C**) The fresh weight. (**D**) MDA staining results under different treatments. The redder the color, the greater the MDA content. Bar = 2 cm. (**E**) H_2_O_2_ staining results under different treatments. The browner the color, the greater the H_2_O_2_ content. Bar = 2 cm. (**F**) O_2_•^−^ staining results under different treatments. The bluer the color, the great the O_2_•^−^ content. Bar = 2 cm. (**G**) MDA content in roots. (**H**) H_2_O_2_ content in roots. (**I**) O_2_•^−^ content in roots. (**J**) SOD activity. (**K**) POD activity. (**L**) CAT activity. (**M**) APX activity. (**N**) GSH content. (**O**) ASA content. Values are the mean ± SD (*n* = 3). Different letters (a–d) in Figure 4 indicate significant differences at *p* < 0.05 according to one-way ANOVA (comparing the mean of each column with the mean of every other column) in GraphPad Prism 7.04.

The expression levels of *CqEXPA50* in quinoa roots and shoots after transient overexpression of *CqEXPA50* were 5- and 16.1-fold that of transient overexpression of the empty vector (Figure S6A). Salt treatment severely affected quinoa development (Figure 4A), significantly inhibiting its root length (Figure 4B) and fresh weight (Figure 4C). Under salt stress, compared with the transient overexpression empty vector, the transient overexpression of *CqEXPA50* improved the development of quinoa seedlings, and the root length and fresh weight increased significantly by 35.6% and 87.3%, respectively (Figure 4A–C). Under salt stress, compared with the transient overexpression empty vector, the chlorophyll and carotenoid contents in quinoa shoots with transient overexpression of *CqEXPA50* increased significantly by 174.4% and 196.6%, respectively (Figure S6B,C).

### 2.10. Lipid Peroxidation and ROS of Quinoa Transiently Overexpressing CqEXPA50

Under salt stress, ROS damage and lipid peroxidation in quinoa roots were very serious, and the roots were stained darker (Figure 4D–F). After transient overexpression of the empty vector, the color of the roots remained dark, while transient overexpression of *CqEXPA50* attenuated root damage (Figure 4D–F). In addition, salt treatment significantly increased the contents of MDA, H_2_O_2_ and O_2_•^−^ in the roots (Figure 4G–I). Under salt stress, compared with the transient overexpression of the empty vector, the MDA, H_2_O_2_ and O_2_•^−^ contents in quinoa roots after transient overexpression of *CqEXPA50* were significantly decreased by 51.4%, 76.2% and 50.3%, respectively (Figure 4G–I). The contents of MDA (Figure S6D), H_2_O_2_ (Figure S6E) and O_2_•^−^ (Figure S6F) in shoots were similar to those in roots (Figure 4G–I).

### 2.11. Antioxidant Enzyme Activities of Quinoa Transiently Overexpressed with CqEXPA50

Compared with the control, the superoxide dismutase (SOD), POD, CAT and ascorbate peroxidase (APX) activity in quinoa roots under salt stress was significantly decreased by 42%, 64.1%, 40.3% and 65.1%, respectively (Figure 4J–M). Under salt stress, compared with the transient overexpression of the empty vector, the SOD, POD, CAT and APX activities in quinoa roots after transient overexpression of *CqEXPA50* were significantly increased by 96.9%, 80.3%, 113.6% and 332.6%, respectively (Figure 4J–M). The antioxidant enzyme activities in shoots (Figure S6G–J) under salt stress were similar to those in roots (Figure 4J–M).

### 2.12. GSH and ASA Contents of Quinoa Transiently Overexpressing CqEXPA50

The GSH and ASA contents in roots were significantly decreased under salt treatment compared to the control (Figure 4N,O). Under salt stress, compared with the transient overexpression of the empty vector, the GSH and ASA contents in roots after transient overexpression of *CqEXPA50* were significantly increased by 86.5% and 57%, respectively (Figure 4N,O). Furthermore, the GSH and ASA contents in shoots (Figure S6K,L) were similar to those in roots (Figure 4N,O).

### 2.13. Systematic Analysis of Auxin Synthesis Pathway Gene Families

To explore the potential mechanism of *CqEXPA50* responding to auxin-mediated salt stress, we comprehensively identified the auxin synthesis pathway gene families from the quinoa genome to screen the genes affected by *CqEXPA50*. A total of 30 *Chenopodium quinoa* auxin response factor (*CqARF*), 41 *Chenopodium quinoa* auxin/indoleacetic acid (*CqAux/IAA*), 18 *Chenopodium quinoa* Gretchen Hagen 3 (*CqGH3*) and 109 *Chenopodium quinoa* small auxin upregulated RNA (*CqSAUR*) genes were identified (Tables S2–S5). Although the CqARF family was identified in previous reports [27], to better identify the target *CqARFs*, we reanalyzed this family with different methods. Their pIs and MWs were predicted (Tables S2–S5). ML phylogenetic trees were constructed with 30 CqARF amino acid sequences and 22 AtARF amino acid sequences, 41 CqIAA amino acid sequences and 25 AtIAA amino acid sequences, 18 CqGH3 amino acid sequences and 20 AtGH3 amino acid sequences, 109 CqSAUR amino acid sequences and 79 AtSAUR amino acid sequences (Figures S7A, S8A, S9A and S10A). These families were further divided into distinct subgroups. All CqARFs contained motifs 1, 4 and 7 and gene structure analysis showed that they all contained conserved domains. The number of introns ranged from 1 to 15 (Figure S7B, Table S7). Most CqAUX/IAAs contain motif 2 with an intron range of 0–15 (Figure S8B, Table S8). Most CqGH3s contain motifs 1 and 2 with introns ranging from 0–6 (Figure S9B, Table S9). All CqSAURs contained motif 2 with an intron range of 0–4 (Figure S10B, Table S10).

Gene duplication analysis found that *CqARFs* had no tandem duplicated genes but one segmental duplicated gene pair (Figure S7C,D, Table S12). *CqAUX/IAAs* had eight pairs of tandem duplicated genes and three pairs of segmental duplicated genes (Figure S8C,D, Table S13). *CqGH3s* had four pairs of tandem duplicated genes and one pair of segmental duplicated genes (Figure S9C,D, Table S14). In contrast, *CqSAURs* had 37 tandem duplicated gene pairs and 10 segmental duplicated pairs (Figure S10C,D, Table S15).

The syntenic analysis found that *CqARFs* did not form a syntenic relationship with rice (*Oryza sativa*) and had the most syntenic gene pairs with beet (*Beta vulgaris*) (Figure S7E, Table S17). *CqIAAs* had syntenic gene pairs with 6 plants, with the most syntenic gene pairs detected in soybean (*Glycine max*) (Figure S8E, Table S18). *CqGH3s* also formed syntenic gene pairs with all 6 plants (Figure S9E, Table S19). *CqSAURs* formed the most syntenic gene pairs with soybean (*Glycine max*) (Figure S10E, Table S20).

### 2.14. CqEXPA50 Mediates Salt Tolerance in Quinoa by Regulating the Auxin Synthesis Pathway and Salt Stress-Related Genes

**Figure 5:**
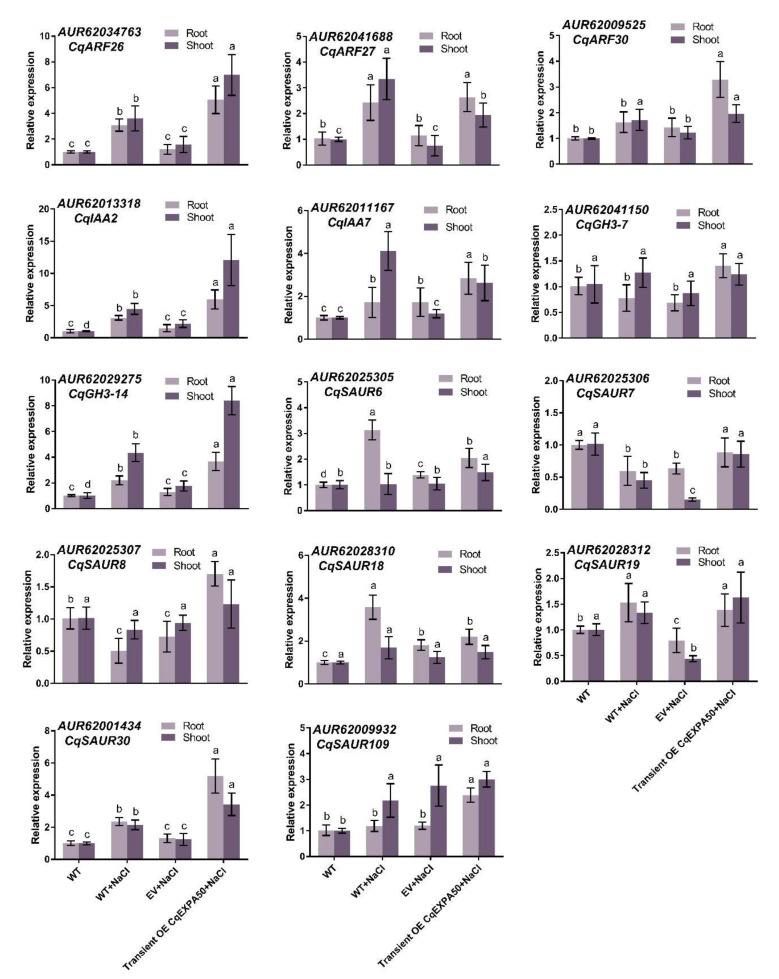
Effects of *CqEXPA50* on the expressions of auxin pathway genes in quinoa root and shoot under salt stress. The expression of auxin pathway genes of all these quinoa seedlings in different treatments were then determined. Values are the mean ± SD (*n* = 3). Different letters (a–d) in Figure 5 indicate significant differences at *p* < 0.05 according to one-way ANOVA (comparing the mean of each column with the mean of every other column) in GraphPad Prism 7.04.

To identify auxin pathway genes regulated by *CqEXPA50*, we determined the expression levels of genes homologous to the *Arabidopsis thaliana* auxin genes in roots and shoots of quinoa transiently overexpressing *CqEXPA50* under salt treatment. These homologous genes responded differently under different treatments (Figure 5). Under salt stress, the expression levels of *CqARF26* (AUR62034763), *CqIAA2* (AUR62013318), *CqGH3-14* (AUR62029275) and *CqSAUR30* (AUR62001434) in quinoa roots after transient overexpression of *CqEXPA50* were 4.2-, 4-, 2.8- and 3.9-fold that of the transient overexpression empty vector, respectively (Figure 5). Under salt stress, the expression levels of *CqARF26* (AUR62034763), *CqIAA2* (AUR62013318), *CqGH3-14* (AUR62029275) and *CqSAUR30* (AUR62001434) in quinoa shoots after transient overexpression of *CqEXPA50* were 4.4-, 5.5-, 4.8- and 2.7-fold that of the transient overexpression empty vector, respectively (Figure 5). Moreover, under salt stress, the expression levels of *Chenopodium quinoa* high-affinity potassium transporter 1 (*CqHKT1*, AUR62027136), *Chenopodium quinoa* Calcineurin B-like 10 (*CqCBL10*, AUR62036054) and *Chenopodium quinoa* Na^+^/H^+^ antiporter 4 (*CqNHX4*, AUR62005035) in quinoa roots after transient overexpression of *CqEXPA50* were 3.9-, 4.9- and 5.4-fold that of the transient overexpression empty vector, respectively (Figure S11). Under salt stress, the expression levels of *CqHKT1* (AUR62027136), *CqCBL10* (AUR62036054) and *CqNHX4* (AUR62005035) in quinoa shoots after transient overexpression of *CqEXPA50* were 9.6-, 5.9- and 6.1-fold that of the transient overexpression empty vector, respectively (Figure S11). Therefore, *CqEXPA50* may affect salt tolerance by regulating the expression of auxin pathway genes (*CqARF26*, *CqIAA2*, *CqGH3-14* and *CqSAUR30*) and salt stress-related genes (*CqHKT1*, *CqCBL10* and *CqNHX4*).

### 2.15. CqEXPA50 Interacts with CqARF26, CqIAA2, CqGH3-14, CqSAUR30, CqHKT1, CqCBL10 and CqNHX4 Proteins

**Figure 6:**
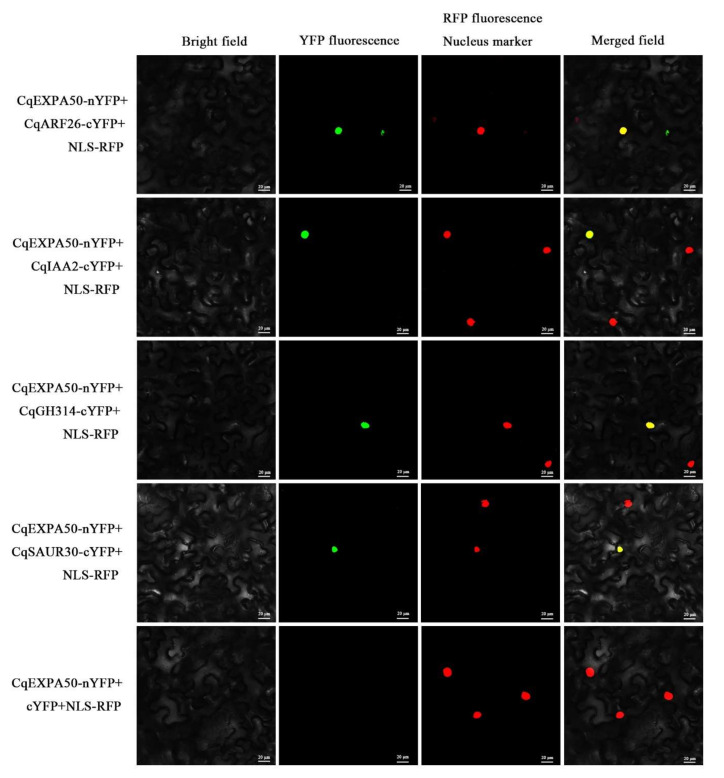
Interactions between CqEXPA50 and CqARF26, CqIAA2, CqGH3-14 and CqSAUR30. CqEXPA50 interact with CqARF26, CqIAA2, CqGH3-14 and CqSAUR30 in *N. benthamiana* leaves. CqEXPA50 was fused with the N-terminal fragment (YN) of yellow fluorescence protein (YFP) to form CqEXPA50-*YN*. CqARF26, CqIAA2, CqGH3-14 and CqSAUR30 were fused with C-terminal fragment of YFP (YC) to form CqARF26-*YC*, CqIAA2-*YC*, CqGH3-14-*YC* and CqSAUR30-*YC*. Green indicates a positive interaction signal. No signal was observed from negative controls. Red represents the nuclear localization signal.

**Figure 7:**
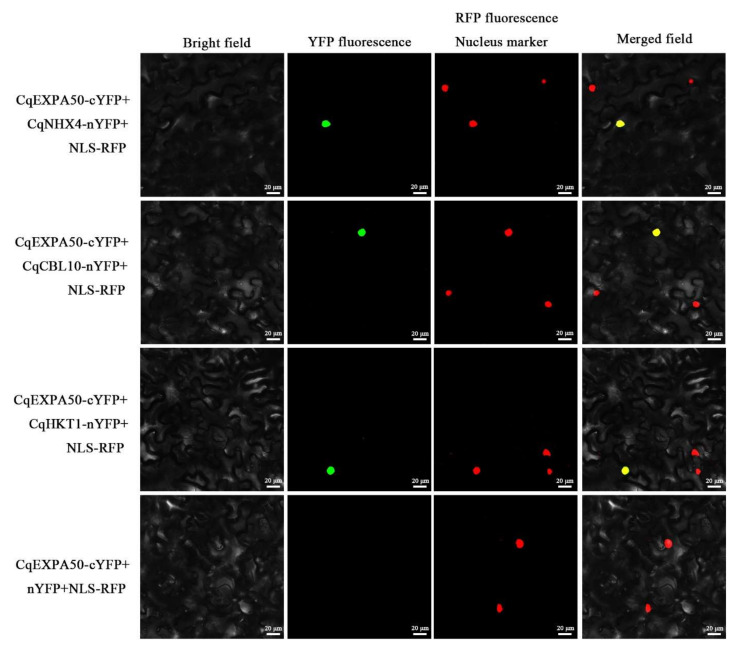
Interactions between CqEXPA50 andCqNHX4, CqCBL10 and CqHKT1. CqEXPA50 interact with CqNHX4, CqCBL10 and CqHKT1 in *N. benthamiana* leaves. CqEXPA50 was fused with the C-terminal fragment (YC) of yellow fluorescence protein (YFP) to form CqEXPA50-*YC*. CqNHX4, CqCBL10 and CqHKT1 were fused with N-terminal fragment of YFP (YN) to form CqNHX4-*YN*, CqCBL10-*YN* and CqHKT1-*YN*. Green indicates a positive interaction signal. No signal was observed from negative controls. Red represents the nuclear localization signal.

Bimolecular fluorescence complementation (BIFC) was used to determine whether CqEXPA50 interacts with the CqARF26, CqIAA2, CqGH3-14, CqSAUR30, CqHKT1, CqCBL10 or CqNHX4 proteins. When CqEXPA50 nYFP and CqARF26 cYFP were coexpressed, a fluorescent signal was observed in tobacco leaf cells, overlapping the nuclear localized fluorescent signal, but not in the negative control (Figure 6). The interaction of CqEXPA50 and CqARF26 occurs in the nucleus. Moreover, CqARF26 can also interact with CqIAA2, CqGH3-14, CqSAUR30, CqHKT1, CqCBL10 and CqNHX4 and the interaction occurs in the nucleus (Figure 6 and Figure 7).

### 2.16. CqARF26, CqIAA2, CqGH3-14, CqSAUR30 and CqHKT1 Contribute to Salt Tolerance in Quinoa

**Figure 8:**
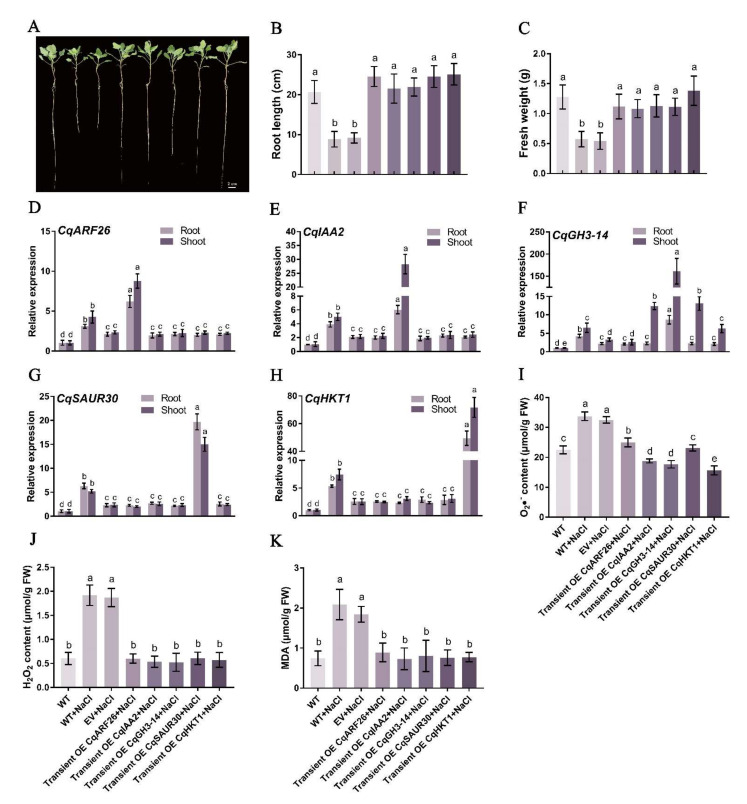
Effect of *CqARF26*, *CqIAA2*, *CqGH3-14*, *CqSAUR30* and *CqHKT1* on salt tolerance in quinoa seedling roots. Relevant indicators of all these quinoa seedling roots in different treatments were determined. (**A**) The effects of *CqARF26*, *CqIAA2*, *CqGH3-14*, *CqSAUR30* and *CqHKT1* on the phenotype of quinoa seedlings under salt stress. Bar = 2 cm. (**B**) The root length. (**C**) The fresh weight. (**D**) Expression analysis of *CqARF26* in quinoa root and shoot under salt stress after transient overexpression of *CqARF26*. (**E**) Expression analysis of *CqIAA2* in quinoa root and shoot under salt stress after transient overexpression of *CqIAA2*. (**F**) Expression analysis of *CqGH3-14* in quinoa root and shoot under salt stress after transient overexpression of *CqGH3-14*. (**G**) Expression analysis of *CqSAUR30* in quinoa root and shoot under salt stress after transient overexpression of *CqSAUR30*. (**H**) Expression analysis of *CqHKT1* in quinoa root and shoot under salt stress after transient overexpression of *CqHKT1*. (**I**) The O2•^−^ content changes in quinoa roots under different treatments. (**J**) The H_2_O_2_ content changes in quinoa roots under different treatments. (**K**) The MDA content changes in quinoa roots under different treatments. Values are the mean ± SD (*n* = 3). Different letters (a–e) in Figure 8B−K indicate significant differences at *p* < 0.05 according to one-way ANOVA (comparing the mean of each column with the mean of every other column) in GraphPad Prism 7.04.

It is necessary to explore whether genes interacting with CqEXPA50 are also involved in salt tolerance. Since the roles of *CqCBL10* and *CqNHX4* in quinoa salt tolerance have been preliminarily explored, their functions were not further explored in this study (unpublished). Under salt stress, the expression levels of these genes in quinoa roots after transient overexpression of *CqARF26*, *CqIAA2*, *CqGH3-14*, *CqSAUR30* and *CqHKT1* were 2.9-, 2.9-, 3.9-, 8.5- and 19.3-fold that of the transient overexpression of the empty vector, respectively (Figure 8D–H). Under salt stress, the expression levels of these genes in quinoa shoots after transient overexpression of *CqARF26*, *CqIAA2*, *CqGH3-14*, *CqSAUR30* and *CqHKT1* were 3.7-, 13.2-, 49.6-, 6.3- and 28.2-fold that of the transient overexpression of the empty vector, respectively (Figure 8D–H). Under salt stress, compared with the transient overexpression empty vector, the transient overexpression of *CqARF26*, *CqIAA2*, *CqGH3-14*, *CqSAUR30* and *CqHKT1* improved quinoa development (Figure 8A). The root length significantly increased by 167%, 134%, 138.4%, 167% and 173.2%, respectively (Figure 8B); the fresh weight significantly increased by 106.3%, 99.7%, 108.4%, 105.4% and 154.9%, respectively (Figure 8C); the chlorophyll content in leaves significantly increased by 43%, 90.8%, 28.4%, 39.3% and 29.8%, respectively (Figure S12A); the carotenoid content in leaves significantly increased by 104.3%, 88.7%, 76.5%, 195.5% and 107.5%, respectively (Figure S12B); the O_2_•^−^ content in roots significantly increased by 23.1%,42.1%, 45.6%, 28.7% and 51.8%, respectively (Figure 8I); the H_2_O_2_ content in roots significantly increased by 67.8%, 71.4%, 71.9%, 67.6% and 69.3%, respectively (Figure 8J); and the MDA content in roots significantly increased by 51.7%, 60.4%, 56.4%, 58.8% and 58%, respectively (Figure 8K). The contents of O_2_•^−^, H_2_O_2_ and MDA in quinoa shoots (Figure S12C–E) were similar to those in roots (Figure 8I,J). Thus, it was found that *CqARF26*, *CqIAA2*, *CqGH3-14*, *CqSAUR30* and *CqHKT1* may be involved in the salt tolerance of quinoa seedlings.

### 2.17. Simultaneously, Transient Overexpression Promotes Salt Tolerance in Quinoa

**Figure 9:**
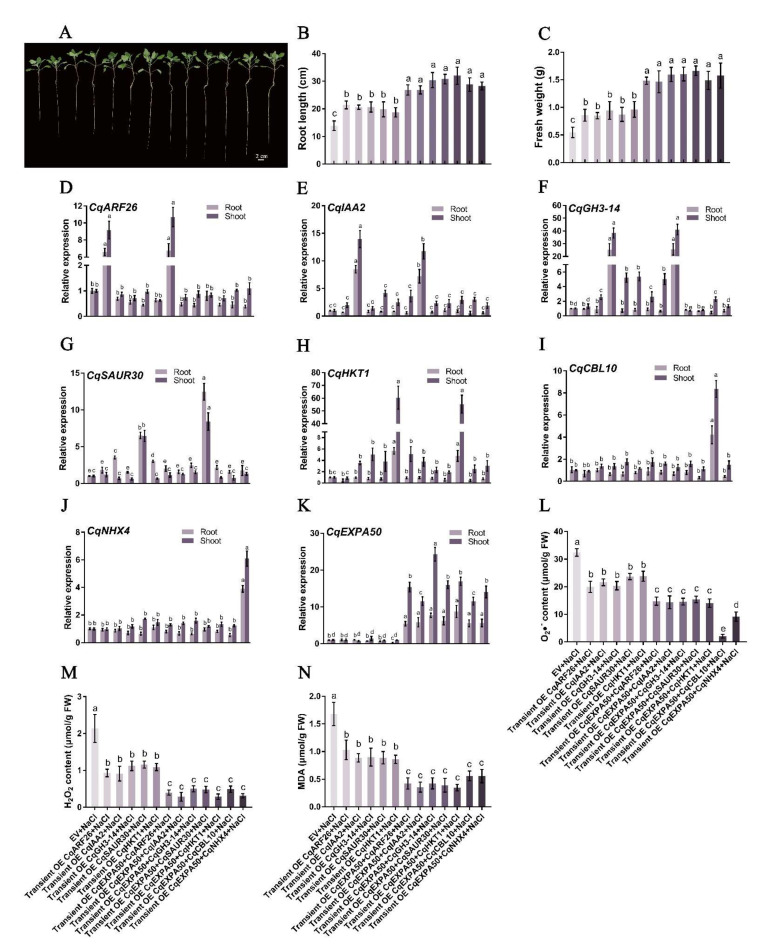
CqEXPA50 participates in salt tolerance of quinoa seedling roots together with CqARF26, CqIAA2, CqGH3-14, CqSAUR30, CqHKT1, CqCBL10 or CqNHX4. Relevant indicators of all these quinoa seedlings roots in different treatments were determined. (**A**) The effects of simultaneous transient overexpression on the phenotype of quinoa seedlings under salt stress. Bar = 2 cm. (**B**) The root length. (**C**) The fresh weight. (**D**) Expression analysis of *CqARF26* in quinoa root and shoot under salt stress after transient overexpression. (**E**) Expression analysis of *CqIAA2* in quinoa root and shoot under salt stress after transient overexpression. (**F**) Expression analysis of *CqGH3-14* in quinoa roots under salt stress after transient overexpression. (**G**) Expression analysis of *CqSAUR30* in quinoa roots under salt stress after transient overexpression. (**H**) Expression analysis of *CqHKT1* in quinoa roots under salt stress after transient overexpression. (**I**) Expression analysis of *CqCBL10* in quinoa roots under salt stress after transient overexpression. (**J**) Expression analysis of *CqNHX4* in quinoa roots under salt stress after transient overexpression. (**K**) Expression analysis of *CqEXPA50* in quinoa roots under salt stress after transient overexpression. (**L**) The O2•^−^ content changes in quinoa roots under different treatments. (**M**) The H_2_O_2_ content changes in quinoa roots under different treatments. (**N**) The MDA content changes in quinoa roots under different treatments. Values are the mean ± SD (*n* = 3). Different letters (a–e) in Figure 9B−N indicate significant differences at *p* < 0.05 according to one-way ANOVA (comparing the mean of each column with the mean of every other column) in GraphPad Prism 7.04.

CqEXPA50 can resist salt stress, and the genes interacting with it are also involved in salt tolerance, so we speculate that CqEXPA50 may enhance the salt tolerance of quinoa when it interacts with these genes. To test this hypothesis, we simultaneously transiently overexpressed *CqEXPA50* and its interacting genes into quinoa to explore their combined effects on salt tolerance. Compared with the transient overexpression of the empty vector, the expression levels of these genes in roots and shoots were significantly increased after simultaneous transient overexpression of *CqEXPA50* and its interacting genes (Figure 9D–K). Under salt stress, compared with transient overexpression of *CqARF26* alone, simultaneous transient overexpression of *CqEXPA50* and *CqARF26* incrementally improved quinoa development (Figure 9A). The root length and fresh weight (Figure 9B,C) were significantly increased by 25.2% and 72.8%, the contents of chlorophyll and carotenoid in the roots were significantly increased by 35.7% and 73.8% (Figure S13A,B), while the contents of O_2_•^−^, H_2_O_2_ and MDA in the roots were significantly decreased by 26.2%, 57.2% and 59%, respectively (Figure 9L–N). Under salt stress, compared with transient overexpression of *CqIAA2* alone, simultaneous transient overexpression of *CqEXPA50* and *CqIAA2* incrementally improved quinoa development (Figure 9A). Root length and fresh weight (Figure 9B,C) were significantly increased by 30.4% and 72.2%, the contents of chlorophyll and carotenoid in roots were significantly increased by 39.4% and 62.2% (Figure S13A,B), while the contents of O_2_•^−^, H_2_O_2_ and MDA in roots were significantly decreased by 33.6%, 68.8% and 59.8%, respectively (Figure 9L–N). The contents of O_2_•^−^, H_2_O_2_ and MDA in shoots (Figure S13C–E) were similar to those in roots (Figure 9L–N).

Similarly, the simultaneous transient transformation of *CqEXPA50* and *CqGH3-14*, *CqEXPA50* and *CqSAUR30* and *CqEXPA50* and *CqHKT1* into quinoa was more effective in maintaining the antioxidant and photosynthetic capacity of quinoa seedlings under salt stress than any of these genes alone (Figure 9 and Figure S13).

## 3. Discussion

### 3.1. Auxin Can Alleviate the Damage of Salt Stress on Quinoa Seedlings

Salt stress is recognized as a key global environmental problem limiting crop yield and development [28], which is further exacerbated by industrial pollution and population growth [29]. Auxin, as a small chemical, plays a key role in plant development and tolerance to environmental stresses [30]. Exogenous application of IAA can confer salt tolerance in faba bean (*Vicia faba* L.) [31]. In the present study, salt stress inhibited the root length and fresh weight of quinoa seedlings, while exogenous IAA alleviated seedling development under salt stress (Figure 1A). In the current study, the photosynthetic pigment contents of quinoa were significantly decreased under salt stress, while IAA promoted their accumulation, indicating that IAA could improve the salt tolerance of quinoa by alleviating the decrease in photosynthetic capacity under salt stress (Figure S3A,B). Salt stress severely reduced chlorophyll content in mustard, while exogenous IAA improved photosynthetic capacity and chlorophyll content under salt stress [32]. ROS causes severe oxidative damage to proteins and nucleic acids, which in turn damages cells and disrupts plant metabolism [33]. Salt stress can promote the accumulation of ROS in plants, increase the level of MDA, disrupt membrane function and lead to cell death [34]. Salt treatment significantly increased O_2_•^−^, H_2_O_2_ and MDA contents in quinoa seedlings (Figure 1 and Figure S3). Previous reports revealed that alfalfa (*Medicago sativa* L.) also accumulated excess MDA and H_2_O_2_ under salt stress [35]. Exogenous application of IAA reduced the accumulation of O_2_•^−^, H_2_O_2_ and MDA in quinoa seedlings under salt stress (Figure 1 and Figure S3), which is consistent with the results observed in other plants [36]. Furthermore, exogenous application of IAA significantly increased the POD and CAT antioxidant enzyme activities of quinoa under salt treatment (Figure 1). There are similar reports that IAA significantly promotes the accumulation of POD enzymatic activities in potato (*Solanum tuberosum* L.) and ground nutrients under salt stress [12]. The current results are consistent with previous reports, suggesting that increased antioxidant enzyme activity contributes to plant tolerance to stress [37,38]. However, with the addition of the auxin inhibitor NPA, the levels of photosynthetic pigments, ROS and enzyme activities in quinoa seedlings were not significantly different from those under salt stress, which further demonstrated that the regulation of antioxidant enzymatic and nonenzymatic systems during the maintenance of quinoa salt tolerance is dependent on IAA.

### 3.2. CqEXPA50 May Be Involved in IAA-Mediated Salt Tolerance

As a key regulator of plant growth and development, auxin plays a major role in regulating cell elongation [39]. Auxin was reported to induce the expression of expansin in pine seedlings [40]. Expansins regulate cell wall extensibility and can alleviate cellular water pressure under adverse conditions and enhance plant stress tolerance [41,42]. As a cell wall-loosening protein, it can increase the exchange of ions and molecules through the cell wall, altering cell physiology and metabolic activity [20]. Expansions play an indispensable role in multiple abiotic stresses. For example, *PttEPA8* in Chinese white poplar (*Populus tomentosa*) plays a key role in the resistance to stresses such as salt, cold and drought [43]. Expansins have been reported to regulate cell wall loosening against adversity stress in *Arabidopsis thaliana* [44], maize (*Zea mays* L.) [45], wheat (*Triticum aestivum* L.) [46] and soybean (*Glycine max*) [47]. Studies have shown that *AtEXP3* (AT2G37640) in *Arabidopsis thaliana* plays a critical role in the salt stress response [48]. Moreover, another report claimed that overexpression of *OsEXPA7* in rice could contribute to salt stress tolerance [22].

We comprehensively identified 78 *Cqexpansins* from quinoa (Figure 2). To identify auxin-mediated salt stress-responsive *Cqexpansins*, we selected genes homologous to *AtEXP3* and measured their expression levels in response to salt, IAA and NPA treatments. *CqEXPA50* was rapidly upregulated under salt treatment, and its expression decreased after adding IAA, and then increased after adding NPA (Figure 3). Therefore, we speculate that it may be involved in IAA-mediated salt tolerance. It is difficult to achieve the stable transformation of quinoa, so *CqEXPA50* was transiently transformed into quinoa to explore its function. This approach has also been widely used in other studies [49]. Consistent with this speculation, transient overexpression of *CqEXPA50* significantly enhanced quinoa salt tolerance. Photosynthetic pigment, ROS and antioxidant enzyme activities in transient *CqEXPA50* overexpression and wild-type quinoa seedlings were determined to explore how *CqEXPA50* participates in salt stress at the physiological and biochemical levels. It was found that *CqEXPA50* could improve the salt tolerance of quinoa by promoting photosynthetic pigment accumulation and maintaining enzymatic and nonenzymatic antioxidant systems. Previous reports have also presented similar results. For instance, overexpression of *AtEXPB2* in tobacco (*Nicotiana tabacum* L.) enhances its ability to resist salt stress by increasing antioxidant enzyme activity and chlorophyll and proline contents [50]. Similarly, overexpression of wheat *TaEXPA2* in tobacco (*Nicotiana tabacum* L.) enhances salt tolerance by improving the chlorophyll content and root development under salt stress [51].

### 3.3. CqEXPA50 Promotes Salt Tolerance of Quinoa through Interactions with Auxin Pathway Genes

Auxin pathway genes, including *ARF*, *AUX/IAA*, *GH3* and *SAUR*, have been reported to play critical roles in the tolerance to various stresses [52,53,54,55]. It has been reported that *AtARF2* (AT5G62000) [56], *AtIAA29* (AT4G32280) [57], *DFL1* (GH3, AT5G54510) [58] and *AtSAUR76* (AT5G20820) [59] play key roles in promoting cell growth and auxin synthesis. The work presented here demonstrates that auxin promotes salt tolerance in quinoa. To explore how *CqEXPA50* is involved in auxin-mediated salt tolerance, we systematically identified the CqARF, CqIAA, CqGH3 and CqSAUR families from the quinoa genome. Combined with expression profiling analysis, it was found that *CqARF26*, *CqIAA2*, *CqGH3-14* and *CqSAUR30* are key genes affected by *CqEXPA50* (Figure 5). Moreover, *CqEXPA50* also significantly induced the expression of the salt stress-related genes *CqHKT1*, *CqCBL10* and *CqNHX4* under salt stress (Figure S11). BIFC further confirmed that CqEXPA50 directly interacts with CqARF26, CqIAA2, CqGH3-14, CqSAUR30, CqHKT1, CqCBL10 and CqNHX4 (Figure 6 and Figure 7). The functions of *CqCBL10* and *CqNHX4* in salt tolerance have been revealed in our other study (unpublished). Since the functions of *CqARF26*, *CqIAA2*, *CqGH3-14*, *CqSAUR30* and *CqHKT1* in quinoa have not yet been explored, we transiently transformed them into quinoa to verify their roles in salt tolerance.

The findings indicate that *CqARF26*, *CqIAA2*, *CqGH3-14*, *CqSAUR30* and *CqHKT1* can also enhance the salt tolerance of quinoa by promoting the accumulation of photosynthetic pigments and reducing antioxidant damage under salt stress (Figure 8 and Figure S11). These results are similar to those of previous reports. It was found that the sweet potato *(Ipomoea batatas*) *IbARF5* gene can promote the tolerance of *Arabidopsis thaliana* to salt stress and drought by regulating carotenoid synthesis, affecting ROS and maintaining antioxidant enzyme activities [60]. It has been reported that the expression of rice *OsIAA9* is significantly induced under salt and drought treatments [61], while overexpression of *OsIAA18* in *Arabidopsis thaliana* significantly promotes salt and osmotic stress tolerance [62]. Virus-induced gene silencing (VIGS) and RT–qPCR experiments also revealed that the cotton *GH3.5* gene is involved in salt and drought tolerance by affecting chlorophyll content, MDA content and SOD enzyme activity [63]. Another study reported that the *Arabidopsis thaliana SAUR41* gene plays a critical role in regulating cell expansion and salt tolerance [64]. More importantly, the simultaneous transient overexpression of *CqEXPA50* in quinoa with auxin genes or salt stress genes further enhanced quinoa salt tolerance, implying their joint involvement in salt tolerance (Figure 9 and Figure S12). However, the functions of these genes still need to be further explored, and the gene regulatory network of quinoa salt stress also needs to be further expanded.

## 4. Materials and Methods

### 4.1. Plant Cultivation and Salt Stress

Quinoa variety Qingbaili 1 seeds were incubated at 25 °C with 16 h of light and 8 h of darkness. Germinated quinoa seedlings were cultured with Hoagland nutrient solution (each liter of Hoagland nutrient solution contains 945 mg Ca(NO_3_)_2_·4H_2_O, 607 mg KNO_3_, 115 mg NH_4_H_2_PO_4_, 493 mg MgSO_4_·7H_2_O, 40 mg [-CH_2_N(CH_2_COONa)CH_2_COO]_2_Fe, 2.86 mg H_3_BO_3_, 2.13 mg MnSO_4_·4H_2_O, 0.22 mg ZnSO_4_·7H_2_O, 0.08 mg CuSO_4_·5H_2_O and 0.02 mg (NH_4_)_6_Mo_7_O_24_·4H_2_O). Quinoa seedlings with similar growth morphology were used for the experiments. In a preliminary experiment, we found that salt stress inhibited the growth of quinoa seedlings (unpublished). Based on a previous result, we chose 150 mM NaCl to simulate salt stress. To explore the alleviation effect of different concentrations of IAA on NaCl, a concentration gradient (0 μM, 1 μM, 3 μM, 5 μM, 7 μM, 10 μM and 15 μM) of IAA was set [65]. Referring to a previous report, a concentration gradient (0 μM, 3 μM, 5 μM, 7 μM and 10 μM) of NPA was set [66]. Twenty quinoa seedlings were used for each treatment group. The Hoagland nutrient solution was replaced every three days. The IAA and NPA concentrations for subsequent experiments were determined by measuring the root length and fresh weight of the quinoa seedlings after two weeks. Three biological replicates were performed.

For subcellular localization and BIFC experiments, 3- to 4-week-old tobacco (*Nicotiana tabacum* L.) seedlings that were grown under the same conditions as quinoa were used.

### 4.2. Determination of Photosynthetic Pigment Content

The total chlorophyll of quinoa seedling leaves in each treatment group was extracted with a mixture of ethanol, acetone and distilled water in a volume ratio of 4.5:4.5:1 [67,68]. The extraction product was kept in the dark. Chlorophyll a, chlorophyll b and carotenoids were detected at 663 nm, 645 nm and 470 nm, respectively. Three biological replicates were performed.

### 4.3. Determination of MDA, H_2_O_2_ and O_2_•^−^ Content and Staining Analysis

The distribution of MDA in roots was determined by Schiff’s reagent staining. Roots and leaves (0.2 g) were extracted with 0.25% 2-thiobarbituric acid (TBA), and the difference in absorption peaks at 532 nm and 600 nm was used to determine the MDA content [69]. Roots were stained with diaminobenzidine (DAB) for H_2_O_2_. Each sample (0.2 g) was ground with 2 mL of 0.1% trichloroacetic acid (TCA) and centrifuged at 12,000 rpm for 8 min, and the supernatant was used to measure the H_2_O_2_ content [70]. The reaction solution for measuring the H_2_O_2_ content was composed of supernatant, enzyme extract, potassium phosphate buffer and KI and the absorbance was measured at 390 nm. The O_2_•^−^ in the roots was stained with nitroblue tetrazolium (NBT) reagent. Each sample (0.2 g) was ground with 2 mL potassium phosphate buffer and centrifuged at 5000 rpm at 4 °C for 8 min [71]. The O_2_•^−^ content was measured at 530 nm. All experiments were performed in three biological replicates.

### 4.4. Determination of Enzyme Activities and GSH and ASA Contents

Roots and leaves (2 g) were ground with 20 mL of phosphate buffer and centrifuged at 5000 rpm at 4 °C for 8 min. The supernatant after centrifugation was assayed for enzyme activity. SOD activity was detected by the photochemical NBT method at 560 nm [72]. POD activity was measured by the increase in absorbance caused by the oxidation of guaiacol at 470 nm [69]. CAT activity can be detected after 60 s of H_2_O_2_ degradation at 240 nm [73]. Potassium phosphate buffer, enzyme extract, ascorbic acid, EDTA-Na_2_ and H_2_O_2_ were mixed and APX activity was detected at 290 nm [74].

Leaves and roots (2 g) were ground with 20 mL of TCA, centrifuged at 12,000 rpm for 8 min and the supernatant was used to determine the GSH and ASA contents. The supernatant, 5,5′-dithiobis-(2-nitrobenzoic acid) (DTNB), NADPH and glutathione reductase were mixed and the GSH content was determined at 412 nm [75]. Then, 30 μL supernatant was mixed with 30 μL dithiothreitol (DTT), incubated at 37 °C for 25 min and then add 15 μL N-ethylmaleimide and 80 μL chromogenic reagent were added, incubated at 37 °C for 45 min and the ASA content was determined at 550 nm [76].

### 4.5. Systematic Identification of Cqexpansin, CqARF, CqIAA, CqGH3 and CqSAUR Families

The quinoa genome was obtained from NCBI. The amino acid sequences of all *expansin*, *ARF*, *IAA*, *GH3* and *SAUR* genes of *Arabidopsis thaliana* were downloaded from the TAIR database. All possible *Cqexpansin, CqARF, CqIAA, CqGH3* and *CqSAUR* genes were identified from the quinoa genome. The amino acid sequences of the *Cqexpansin, CqARF, CqIAA, CqGH3* and *CqSAUR* genes were BLASTP to remove any incorrect genes. Moreover, the conserved domains of the above *Cqexpansin, CqARF, CqIAA, CqGH3* and *CqSAUR* genes were predicted by the Web CD search tool, and any remaining incorrect genes were removed.

### 4.6. Maximum Likelihood (ML) Trees Construction of Cqexpansin, CqARF, CqIAA, CqGH3 and CqSAUR Families

The amino acid sequences of the *expansin*, *ARF*, *IAA* and *GH3* and *SAUR* genes in *Arabidopsis thaliana* and quinoa were aligned in Multiple Sequence Comparison by Log Expectation (MUSCLE) [77] of MEGA 7 [78] to determine the optimal protein model. The best model for the *ARF*, *IAA*, *GH3* and *SAUR* genes in *Arabidopsis thaliana* and quinoa was the Jones–Taylor–Thornton (JTT) model, while the best model for the *expansin* genes in *Arabidopsis thaliana* and quinoa was the Wheeler and Goldman (WAG) model. The best model for *Cqexpansin* and *CqIAA* was WAG. The best model for *CqARF* and *CqSAUR* was JTT. The best model for *CqGH3* was LG. The ML trees were constructed in MEGA 7 [78] using the corresponding best model with 1000 bootstrap replicates.

### 4.7. Gene Structure, Conserved Domains and Motif Composition of the Cqexpansin, CqARF, CqIAA, CqGH3 and CqSAUR Families

Conserved Domain Search Service was used to analyze the conserved domains of the *Cqexpansin*, *CqARF*, *CqIAA*, *CqGH3* and *CqSAUR* genes [79]. The Multiple Expectation maximization for Motif Elicitation (MEME) online tool was used to determine the motif composition of the *Cqexpansin*, *CqARF*, *CqIAA*, *CqGH3* and *CqSAUR* genes [80]. Gene structure prediction of *Cqexpansin*, *CqARF*, *CqIAA*, *CqGH3* and *CqSAUR* was performed with the Gene Structure Display Server online tool [81]. TBtools was used for visualization [82].

### 4.8. Duplication and Localization Analysis of the Cqexpansin, CqARF, CqIAA, CqGH3 and CqSAUR Genes

The chromosomal locations of the *Cqexpansin*, *CqARF*, *CqIAA*, *CqGH3* and *CqSAUR* genes were determined using the quinoa genome sequences and the General Feature Format (GFF) files. The MCScanX toolkit was used to determine the tandem and segment duplication genes among the *Cqexpansin*, *CqARF*, *CqIAA*, *CqGH3* and *CqSAUR* genes [83]. TBtools was used for visualization [82].

### 4.9. Syntenic Analysis of Expansin, ARF, IAA, GH3 and SAUR Genes in Quinoa and Six Plants

BLAST and MCScanX were used to analyze the synteny of the *Cqexpansin*, *CqARF*, *CqIAA*, *CqGH3* and *CqSAUR* genes. The synteny among the *Cqexpansin*, *CqARF*, *CqIAA*, *CqGH3* and *CqSAUR* genes and the *expansin*, *ARF*, *IAA*, *GH3* and *SAUR* genes from other six representative plants were shown with TBtools [82].

### 4.10. Gene Expression Profile Analysis

The first treatment included a control, 150 mM NaCl, 150 mM NaCl + 3 μM IAA and 150 mM NaCl + 3 μM IAA + 7 μM NPA. The second treatment included a control, wild type (WT) + 150 mM NaCl, empty vector + 150 mM NaCl, transiently overexpressed *CqEXPA50* + 150 mM NaCl, transiently overexpressed *CqARF26* + 150 mM NaCl, transiently overexpressed *CqIAA2* + 150 mM NaCl, transiently overexpressed *CqGH3-14* + 150 mM NaCl, transiently overexpressed *CqSAUR30* + 150 mM NaCl, transiently overexpressed *CqHKT1* + 150 mM NaCl, simultaneous transient overexpression of *CqEXPA50* and *CqARF26* + 150 mM NaCl, simultaneous transient overexpression of *CqEXPA50* and *CqIAA2* + 150 mM NaCl, simultaneous transient overexpression of *CqEXPA50* and *CqGH3-14* + 150 mM NaCl, simultaneous transient overexpression of *CqEXPA50* and *CqSAUR30* + 150 mM NaCl, simultaneous transient overexpression of *CqEXPA50* and *CqHKT1* + 150 mM NaCl, simultaneous transient overexpression of *CqEXPA50* and *CqCBL10* + 150 mM NaCl and simultaneous transient overexpression of *CqEXPA50* and *CqNHX4* + 150 mM NaCl. RNA was extracted from quinoa roots and shoots treated for two weeks using an RNA kit. RNA was reverse transcribed to cDNA using the PrimeScript RT reagent Kit (Vazyme, Nanjing, China). The Bio-Rad CFX96 real-time PCR system was used for quantitative real-time polymerase chain reaction (qRT-PCR) experiments. Primers for *Cqexpansin*, *CqARF*, *CqIAA*, *CqGH3* and *CqSAUR* were designed with Primer 3 (Table S21). Elongation factor 1α was used as a reference gene [84]. The relative expression level was calculated as 2^−^^ΔΔCt^ [85].

### 4.11. Transient Overexpression of CqEXPA50, CqARF26, CqIAA2, CqGH3-14, CqSAUR30, CqHKT1, CqCBL10 and CqNHX4 in Quinoa

The CDSs of *CqEXPA50, CqARF26, CqIAA2, CqGH3-14, CqSAUR30, CqHKT1, CqCBL10* and *CqNHX4* were cloned into pCAMBIA1300 and the recombinant plasmids were transformed into Agrobacterium GV3101. Agrobacterium was resuspended to an optical density (OD) of 1 in a buffer containing 10 mM MES-KOH, 10 mM MgCl_2_, and 100 μM acetylsyringone. The suspensions of *CqEXPA50* and *CqARF26*, *CqEXPA50* and *CqIAA2*, *CqEXPA50* and *CqGH3-14*, *CqEXPA50* and *CqSAUR30*, *CqEXPA50* and *CqHKT1*, *CqEXPA50* and *CqCBL10* and *CqEXPA50* and *CqNHX4* were mixed at a ratio of 1:1. The suspensions were injected into the quinoa leaves with a 1 mL needleless syringe, avoiding the vein. Then, the plants were cultivated in the dark for 24 h and then moved into the light. Meanwhile, the roots of the quinoa seedlings were soaked with the same suspension. The quinoa seedlings were repeatedly injected and soaked every five days. Successfully transformed plants were screened for further experiments by measuring the expression levels of *CqEXPA50, CqARF26, CqIAA2, CqGH3-14, CqSAUR30, CqHKT1, CqCBL10* and *CqNHX4*. The various indicators of the quinoa seedlings were measured after two weeks of treatment. Three biological replicates were performed for each experiment.

### 4.12. Subcellular Localization of CqEXPA50

The CDS of *CqEXPA50* was cloned into the pCAMBIA1300 vector. The recombinant plasmid pCAMBIA1300-*CqEXPA50* was transformed into Agrobacterium GV3101. The method for suspending Agrobacterium was the same as the abovementioned preparation method for transient transformation of quinoa. The recombinant plasmid pCAMBIA1300-*CqEXPA50* was introduced into the epidermal cells of tobacco leaves. pCAMBIA1300 was used as a negative control. After transient transformation, plants were cultured in the dark for 24 h and observed under a laser confocal microscope. The GFP fluorescence signals were examined using excitation and emission wavelengths of 488 nm and 500-550 nm, respectively. The GFP channel was selected to visualize the yellow fluorescence.

### 4.13. BIFC

The CDS of *CqEXPA50* was fused with the N-terminal fragment of YFP (YN) to form CqEXPA50-YN. The CDSs of *CqARF26, CqIAA2, CqGH3-14, CqSAUR30, CqHKT1, CqCBL10 and CqNHX4* were fused with the C-terminal fragment (YC) of yellow fluorescence protein (YFP) to form CqARF26-YC, CqIAA2-YC, CqGH3-14-YC, CqSAUR3-YC, CqHKT1-YC, CqCBL10-YC and CqNHX4-YC, respectively. These recombinant plasmids were transformed into Agrobacterium GV3101. The preparation method of the Agrobacterium suspension was the same as above. The *CqEXPA50* suspension was mixed with suspensions of *CqARF26, CqIAA2, CqGH3-14, CqSAUR30, CqHKT1, CqCBL10* and *CqNHX4* at a ratio of 1:1 and incubated in the dark for 2 h. The prepared suspension was injected into tobacco leaves, avoiding the veins, followed by one day of dark culture followed by light culture. Fluorescence signals were observed by laser confocal microscopy. The excitation and emission wavelengths were set the same as for the subcellular localization.

## 5. Conclusions

**Figure 10:**
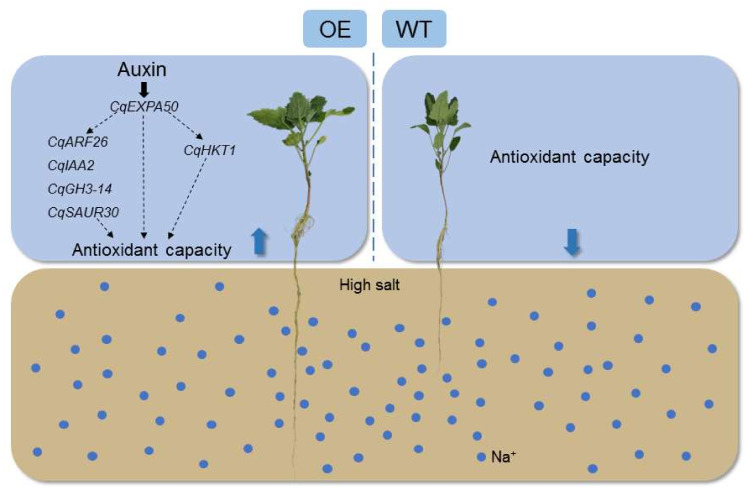
A proposed model to illustrate how *CqEXP50*, *CqARF26*, *CqIAA2*, *CqGH3-14*, *CqSAUR30* and *CqHKT1* enhance salt tolerance of quinoa seedlings.

Salt stress restricts crop yield and threatens the safety of agricultural products. The current study demonstrates that auxin can act as a regulator to alleviate salt stress in quinoa. A comprehensive analysis of the Cqexpansin and auxin pathway gene families (CqARF, CqIAA, CqGH3 and CqSAUR) was performed. Combined expression profiling, transient overexpression and physiological and biochemical analyses confirmed that *CqEXPA50*, *CqARF26*, *CqIAA2*, *CqGH3-14*, *CqSAUR30* and *CqHKT1* could contribute to the salt tolerance of quinoa (Figure 10). Notably, *CqEXPA50* enhanced salt tolerance when cooperating with the auxin pathway and salt stress genes. The candidate genes identified in this study can lay the foundation for the selection and breeding of stress-resistant varieties.

Blue dots represent Na+. The blue arrow up represents improvement and the blue arrow down represents reduction. *CqEXP50*, *CqARF26*, *CqIAA2*, *CqGH3-14*, *CqSAUR30* and *CqHKT1* can improve the antioxidant capacity of quinoa seedlings under salt stress.

## Data Availability

The data presented in this study are available in article and Supplementary Material.

## Supplementary Materials

The following supporting information can be downloaded at: https://www.mdpi.com/article/10.3390/ijms23158480/s1.

## Abbreviations

Ascorbate peroxidase (APX); Ascorbic acid (ASA); Bimolecular fluorescence complementation (BIFC); Catalase (CAT); Chenopodium quinoa auxin response fac-tor (CqARF); Chenopodium quinoa auxin/indoleacetic acid (CqAux/IAA); Chenopodium quinoa Calcineurin B-like 10 (CqCBL10); Chenopodium quinoa expansin (Cqexpansin); Chenopodium quinoa α-expansin (CqEXPA); Chenopodium quinoa Gretchen Hagen 3 (CqGH3); Chenopodium quinoa high-affinity po-tassium transporter 1 (CqHKT1); Chenopodium quinoa Na^+^/H^+^ antiporter 4 (CqNHX4); Chenopodium quinoa small auxin up-regulated RNA (CqSAUR); Diaminobenzidine (DAB); 5,5’-dithiobis-(2-nitrobenzoic acid) (DTNB); Dithiothreitol (DTT); General Feature Format (GFF); Glutathione (GSH); Hydrogen peroxide (H_2_O_2_); Indole-3-acetic acid (IAA); Jones-Taylor-Thornton (JTT); Malondialdehyde (MDA); Multiple Expectation maximization for Motif Elicitation (MEME); Maximum likelihood (ML); Multiple Sequence Comparison by Log Expectation (MUSCLE); Nitroblue tetrazolium (NBT); Nicotiana tabacum α-expansin 11 (NtEXPA11); N-1-naphthylphthalamic acid (NPA); Optical density (OD); Peroxidase (POD); Quantitative real-time polymerase chain reaction (qRT-PCR); Reactive oxygen species (ROS); Superoxide anion (O_2_•^−^); Superoxide dismutase (SOD); 2-thiobarbituric acid (TBA); Trichloroacetic acid (TCA); Virus-induced gene silencing (VIGS); Wheeler and Goldman (WAG); Wild type (WT).
