# Supplementary material for: Involvement of Auxin-Mediated CqEXPA50 Contributes to Salt Tolerance in Quinoa (Chenopodium quinoa) by Interaction with Auxin Pathway Genes
Source: Int J Mol Sci. 2022 Jul 30;23(15):8480. doi: 10.3390/ijms23158480 (PMC9369402; doi:10.3390/ijms23158480)
Supplement: Supplementary file 1 [file ijms-23-08480-s001.zip › Supporting information 7.29.pdf]

Supporting Information:

Figure S1. Alleviating effects of different concentrations of IAA on the growth of quinoa seedlings under salt stress

Quinoa seedlings of six true leaves were cultured in 0, 150 mM NaCl, 150 mM NaCl+1 μM IAA, 150 mM NaCl+3 μM IAA, 150 mM NaCl+5 μM IAA, 150 mM NaCl+7 μM IAA, 150 mM NaCl+10 μM IAA and 150 mM NaCl+15 μM IAA-Hoagland solution for two weeks, and then phenotype, root length and fresh weight were recorded. (A) Phenotypic changes of quinoa seedlings under different salt concentrations; (B) The root length; (C) The fresh weight. Values are mean ± SD (n = 3). Bar = 2 cm. Different letters indicate significant differences at $p < 0.05$ according to one-way ANOVA (comparing the mean of each column with the mean of every other column) in GraphPad Prism 7.04.

Figure S2. Effects of different concentrations of NPA on auxin alleviating salt stress in quinoa seedlings

Quinoa seedlings of six true leaves were cultured in Hoagland solution, 150 mM NaCl-Hoagland solution, 150 mM NaCl+3 μM IAA-Hoagland solution, and 3, 5, 7 and 10 μM NPA with 150 mM NaCl+3 μM IAA-Hoagland solution for two weeks, and then phenotype, root length and fresh weight were recorded. (A) Phenotypic changes of quinoa seedlings under different malic acid concentrations; (B) The root length; (C) The fresh weight. Values are mean ± SD (n = 3). Bar = 2 cm. Different letters indicate significant differences at $p < 0.05$ according to one-way ANOVA (comparing the mean of each column with the mean of every other column) in GraphPad Prism 7.04.

Figure S3. Effect of IAA on the growth, photosynthetic capacity, and antioxidant capacity of quinoa shoots under salt stress

Quinoa seedlings of six true leaves were cultured in Hoagland solution (CK), 150 mM NaCl-Hoagland solution, 150 mM NaCl+3 μM IAA-Hoagland solution and 150 mM NaCl+3 μM IAA+7 μM NPA-Hoagland solution for two weeks. (A) chlorophyll content changes of quinoa seedlings under different treatments; (B) The carotenoids content. (C) $O_2^{\bullet -}$ content in shoots; (D) H_2O_2 content in shoots; (E) MDA content in shoots. (F) POD activity in shoots; (G) CAT activity in shoots. (H) GSH content in shoots; (I) ASA content in shoots. Values are mean ± SD (n = 3). Different letters indicate significant differences at $p < 0.05$ according to one-way ANOVA (comparing the mean of each column with the mean of every other column) in GraphPad Prism 7.04.

Figure S4. Gene structure, chromosomal localization, and synteny analysis of the *Cqexpansin* genes

(A) Phylogenetic relationships, gene structure, conserved domain and conserved protein motifs in expansin genes from quinoa. (i) The maximum likelihood tree was constructed based on the full-length sequences of quinoa expansin genes. (ii) The motif composition of quinoa expansin genes. The motifs, numbers 1–10, are displayed in different colored boxes. (iii) Exon-intron structure and conserved domains of quinoa expansin genes. Black lines indicate introns. The number indicates the phases of the corresponding introns. (B) Schematic representations for the chromosomal distribution of quinoa expansin genes. The red lines indicate duplicated expansin gene pairs. (C) Schematic representations of the interchromosomal relationships of quinoa expansin genes. Grey and blue lines indicate all homology blocks in the quinoa genome, and the blue lines indicate duplicated Cqexpansin gene pairs. (D) Synteny analysis of expansin genes between quinoa and six representative plant species. Gray lines in the background indicate the collinear blocks within quinoa and other plant genomes, while red lines highlight syntenic expansin gene pairs.

Figure S5. The subcellular localization of CqEXPA50.

Agrobacteria carrying CqEXPA50 or control vector YFP were infiltrated into leaves of *N. benthamiana* with the nuclear maker, and the fluorescence images were taken in a dark field for yellow and red fluorescence, in the white field for the morphology of the cell, and in combination. Bright: bright field; YFP: YFP fluorescence; RFP: RFP fluorescence; Merged: YFP/bright/RFP field overlay. Bar = 20 μm.

Figure S6. Effects of CqEXPA50 on the growth, photosynthetic capacity, and antioxidant capacity of quinoa shoots under salt stress.

Quinoa seedlings with six true leaves were cultured in Hoagland solution (control) and 150 mM NaCl-Hoagland solution. Quinoa seedlings with six true leaves that were transiently overexpressed with the empty vector and CqEXPA50 were separately cultured in 150 mM NaCl-Hoagland solution for two weeks. (A) The expression of CqEXPA50 of all these quinoa seedlings in different treatments were then determined. (B) chlorophyll content changes of quinoa seedlings under different treatments. (C) The carotenoids content. (D) MDA content in shoots. (E) H_2O_2 content in shoots. (F) $O_2{\bullet}^-$ content in shoots. (G) SOD activity. (H) POD activity. (I) CAT activity. (J) APX activity. (K) GSH content. (L) ASA content. Values are the mean ± SD (*n* = 3). Different letters indicate significant differences at $p < 0.05$ according to one-way ANOVA (comparing the mean of each column with the mean of every other column) in GraphPad Prism 7.04.

Figure S7. Analysis and identification of CqARF family in quinoa.

(A) Phylogenetic tree construction and subfamily classification of ARF family in quinoa and *Arabidopsis*. Maximum likelihood tree based on the full-length sequences of the 22 *Arabidopsis ARF* genes and 30 quinoa *ARF* genes were constructed under JTT model using Mega 7. (B) Phylogenetic relationships, gene structure, conserved domain and conserved protein motifs in *ARF* genes from quinoa. (i) The maximum likelihood tree was constructed based on the full-length sequences of quinoa *ARF* genes under JTT model using Mega 7. (ii) The motif composition of quinoa *ARF* genes. The motifs, numbers 1–10, are displayed in different colored boxes. The protein length can be estimated using the scale at the bottom. (iii) Exon-intron structure and conserved domains of quinoa *ARF* genes. Black lines indicate introns. The number indicates the phases of the corresponding introns. (C) Schematic representations for the chromosomal distribution of quinoa *ARF* genes. The chromosome number is indicated to the left of each chromosome. (D) Schematic representations of the interchromosomal relationships of quinoa *ARF* genes. Grey and blue lines indicate all homology blocks in the quinoa genome, and the blue lines indicate duplicated *CqARF* gene pairs. (E) Synteny analysis of *ARF* genes between quinoa and six representative plant species. Gray lines in the background indicate the collinear blocks within quinoa and other plant genomes (*Beta vulgaris*, *Glycine max*, *Solanum lycopersicum*, *Fagopyrum tataricum*, *Oryza sativa*, and *Arabidopsis thaliana*), while red lines highlight syntenic *ARF* gene pairs.

Figure S8. Analysis and identification of CqAUX/IAA family in quinoa.

(A) Phylogenetic tree construction and subfamily classification of AUX/IAA family in quinoa and *Arabidopsis*. Maximum likelihood tree based on the full-length sequences of the 25 *Arabidopsis AUX/IAA* genes and 41 quinoa *AUX/IAA* genes were constructed under JTT model using Mega 7. (B) Phylogenetic relationships, gene structure, conserved domain and conserved protein motifs in *AUX/IAA* genes from quinoa. (i) The maximum likelihood tree was constructed based on the full-length sequences of quinoa *AUX/IAA* genes under WAG model using Mega 7. (ii) The motif composition of quinoa *AUX/IAA* genes. The motifs, numbers 1–10, are displayed in different colored boxes. The protein length can be estimated using the scale at the bottom. (iii) Exon-intron structure and conserved domains of quinoa *AUX/IAA* genes. Black lines indicate introns. The number indicates the phases of the corresponding introns. (C) Schematic representations for the chromosomal distribution of quinoa AUX/IAA genes. The red lines indicate duplicated *AUX/IAA* gene pairs. The chromosome number is indicated to the left of each chromosome. (D) Schematic representations of the interchromosomal

relationships of quinoa *AUX/IAA* genes. Grey and blue lines indicate all homology blocks in the quinoa genome, and the blue lines indicate duplicated *CqAUX/IAA* gene pairs. (E) Synteny analysis of *AUX/IAA* genes between quinoa and six representative plant species. Gray lines in the background indicate the collinear blocks within quinoa and other plant genomes (*Beta vulgaris*, *Glycine max*, *Solanum lycopersicum*, *Fagopyrum tataricum*, *Oryza sativa*, and *Arabidopsis thaliana*), while red lines highlight syntenic *AUX/IAA* gene pairs.

Figure S9. Analysis and identification of CqGH3 family in quinoa.

(A) Phylogenetic tree construction and subfamily classification of GH3 family in quinoa and *Arabidopsis*. Maximum likelihood tree based on the full-length sequences of the 20 *Arabidopsis GH3* genes and 18 quinoa *GH3* genes were constructed under JTT model using Mega 7. (B) Phylogenetic relationships, gene structure, conserved domain and conserved protein motifs in *GH3* genes from quinoa. (i) The maximum likelihood tree was constructed based on the full-length sequences of quinoa *GH3* genes under LG model using Mega 7. (ii) The motif composition of quinoa *GH3* genes. The motifs, numbers 1–10, are displayed in different colored boxes. The protein length can be estimated using the scale at the bottom. (iii) Exon-intron structure and conserved domains of quinoa *GH3* genes. Black lines indicate introns. The number indicates the phases of the corresponding introns. (C) Schematic representations for the chromosomal distribution of quinoa *GH3* genes. The red lines indicate duplicated *GH3* gene pairs. The chromosome number is indicated to the left of each chromosome. (D) Schematic representations of the interchromosomal relationships of quinoa *GH3* genes. Grey and blue lines indicate all homology blocks in the quinoa genome, and the blue lines indicate duplicated *CqGH3* gene pairs. (E) Synteny analysis of *GH3* genes between quinoa and six representative plant species. Gray lines in the background indicate the collinear blocks within quinoa and other plant genomes (*Beta vulgaris*, *Glycine max*, *Solanum lycopersicum*, *Fagopyrum tataricum*, *Oryza sativa*, and *Arabidopsis thaliana*), while red lines highlight syntenic *GH3* gene pairs.

Figure S10. Analysis and identification of CqSAUR family in quinoa.

(A) Phylogenetic tree construction and subfamily classification of SAUR family in quinoa and *Arabidopsis*. Maximum likelihood tree based on the full-length sequences of the 79 *Arabidopsis SAUR* genes and 109 quinoa *SAUR* genes were constructed under JTT model using Mega 7. (B) Phylogenetic relationships, gene structure, conserved domain and conserved protein motifs in *SAUR* genes from quinoa. (i) The maximum likelihood tree was constructed based on the full-length sequences of quinoa

SAUR genes under JTT model using Mega 7. (ii) The motif composition of quinoa *SAUR* genes. The motifs, numbers 1–10, are displayed in different colored boxes. The protein length can be estimated using the scale at the bottom. (iii) Exon-intron structure and conserved domains of quinoa *SAUR* genes. Black lines indicate introns. The number indicates the phases of the corresponding introns. (C) Schematic representations for the chromosomal distribution of quinoa *SAUR* genes. The red lines indicate duplicated *SAUR* gene pairs. The chromosome number is indicated to the left of each chromosome. (D) Schematic representations of the interchromosomal relationships of quinoa *SAUR* genes. Grey and blue lines indicate all homology blocks in the quinoa genome, and the blue lines indicate duplicated *CqSAUR* gene pairs. (E) Synteny analysis of *SAUR* genes between quinoa and six representative plant species. Gray lines in the background indicate the collinear blocks within quinoa and other plant genomes (*Beta vulgaris*, *Glycine max*, *Solanum lycopersicum*, *Fagopyrum tataricum*, *Oryza sativa*, and *Arabidopsis thaliana*), while red lines highlight syntenic *SAUR* gene pairs.

Figure 11. Effects of CqEXPA50 on the expressions of salt stress-related genes in quinoa root and shoot under salt stress.

Quinoa seedlings with six true leaves were cultured in Hoagland solution (control) and 150 mM NaCl-Hoagland solution. Quinoa seedlings with six true leaves that were transiently overexpressed with the empty vector and CqEXPA50 were separately cultured in 150 mM NaCl-Hoagland solution for two weeks. Finally, the expression of salt stress-related genes of all these quinoa seedlings in different treatments were then determined. Values are the mean ± SD (n = 3). Different letters indicate significant differences at $p < 0.05$ according to one-way ANOVA (comparing the mean of each column with the mean of every other column) in GraphPad Prism 7.04.

Figure S12. Effect of CqARF26, CqIAA2, CqGH3-14, CqSAUR30 and CqHKT1 on salt tolerance in quinoa seedling shoots

Quinoa seedlings with six true leaves were cultured in Hoagland solution (control) and 150 mM NaCl-Hoagland solution. Quinoa seedlings with six true leaves that were transiently overexpressed with the empty vector, CqARF26, CqIAA2, CqGH3-14, CqSAUR30 and CqHKT1 were separately cultured in 150 mM NaCl-Hoagland solution for two weeks. Finally, relevant indicators of all these quinoa seedling shoots in different treatments were then determined. (A) The chlorophyll content changes of quinoa seedlings under different treatments. Values are the mean ± SD (n = 3). Different letters indicate significant differences at $p < 0.05$ according to Duncan's multiple range test. (B) The carotenoid content

changes of quinoa seedlings under different treatments. Values are the mean ± SD (n = 3). Different letters indicate significant differences at $p < 0.05$ according to Duncan's multiple range test. (C) The $O_2^{\bullet -}$ content changes of quinoa shoots under different treatments. Values are the mean ± SD (n = 3). Different letters indicate significant differences at $p < 0.05$ according to Duncan's multiple range test. (D) The H_2O_2 content changes of quinoa shoots under different treatments. Values are the mean ± SD (n = 3). Different letters indicate significant differences at $p < 0.05$ according to Duncan's multiple range test. (E) The MDA content changes of quinoa shoots under different treatments. Values are the mean ± SD (*n* = 3). Different letters indicate significant differences at $p < 0.05$ according to one-way ANOVA (comparing the mean of each column with the mean of every other column) in GraphPad Prism 7.04.

Figure S13. *CqEXPA50* participates in salt tolerance of quinoa seedling shoots together with *CqARF26, CqIAA2, CqGH3-14, CqSAUR30, CqHKT1, CqCBL10* or *CqNHX4*

Quinoa seedlings with six true leaves that were transiently overexpressed with the empty vector, *CqARF26, CqIAA2, CqGH3-14, CqSAUR30 and CqHKT1*, a mixture of *CqEXPA50* and *CqARF26*, *CqEXPA50 and CqIAA2, CqEXPA50 and CqGH3-14, CqEXPA50 and CqSAUR30, CqEXPA50 and CqHKT1, CqEXPA50* and *CqCBL10* and *CqEXPA50* and *CqNHX4* were separately cultured in 150 mM NaCl-Hoagland solution for two weeks. Finally, relevant indicators of all these quinoa seedlings shoots in different treatments were then determined. (A) The chlorophyll content changes of quinoa seedlings under different treatments. Values are the mean ± SD (n = 3). Different letters indicate significant differences at $p < 0.05$ according to Duncan's multiple range test. (B) The carotenoid content changes of quinoa seedlings under different treatments. Values are the mean ± SD (n = 3). Different letters indicate significant differences at $p < 0.05$ according to Duncan's multiple range test. (C) The $O_2^{\bullet -}$ content changes of quinoa shoots under different treatments. Values are the mean ± SD (n = 3). Different letters indicate significant differences at $p < 0.05$ according to Duncan's multiple range test. (D) The H_2O_2 content changes of quinoa shoots under different treatments. Values are the mean ± SD (n = 3). Different letters indicate significant differences at $p < 0.05$ according to Duncan's multiple range test. (E) The MDA content changes of quinoa shoots under different treatments. Values are the mean ± SD (*n* = 3). Different letters indicate significant differences at $p < 0.05$ according to one-way ANOVA (comparing the mean of each column with the mean of every other column) in GraphPad Prism 7.04.

Supplementary tables

Table S1. List of the 78 *Cqexpansin* genes identified in this study

Table S2. List of the 30 *CqARF* genes identified in this study

Table S3. List of the 41 *CqAUX/IAA* genes identified in this study

Table S4. List of the 18 *CqGH3* genes identified in this study

Table S5. List of the 109 *CqSAUR* genes identified in this study

Table S6. Analysis and distribution of conserved motifs in quinoa *expansin* genes

Table S7. Analysis and distribution of conserved motifs in quinoa *ARF* genes

Table S8. Analysis and distribution of conserved motifs in quinoa *AUX/IAA* genes

Table S9. Analysis and distribution of conserved motifs in quinoa *GH3* genes

Table S10. Analysis and distribution of conserved motifs in quinoa *SAUR* genes

Table S11. Segmental duplicate gene pairs of *Cqexpansin* genes

Table S12. Segmental duplicate gene pairs of *CqARF* genes

Table S13. Segmental duplicate gene pairs of *CqAUX/IAA* genes

Table S14. Segmental duplicate gene pairs of *CqGH3* genes

Table S15. Segmental duplicate gene pairs of *CqSAUR* genes

Table S16. Synteny gene pairs between *Cqexpansin* genes and other plants

Table S14. Synteny gene pairs between *CqARF* genes and other plants

Table S15. Synteny gene pairs between *CqAUX/IAA* genes and other plants

Table S14. Synteny gene pairs between *CqGH3* genes and other plants

Table S15. Synteny gene pairs between *CqSAUR* genes and other plants

Table S21 Primers sequences for RT-qPCR

Figure S1

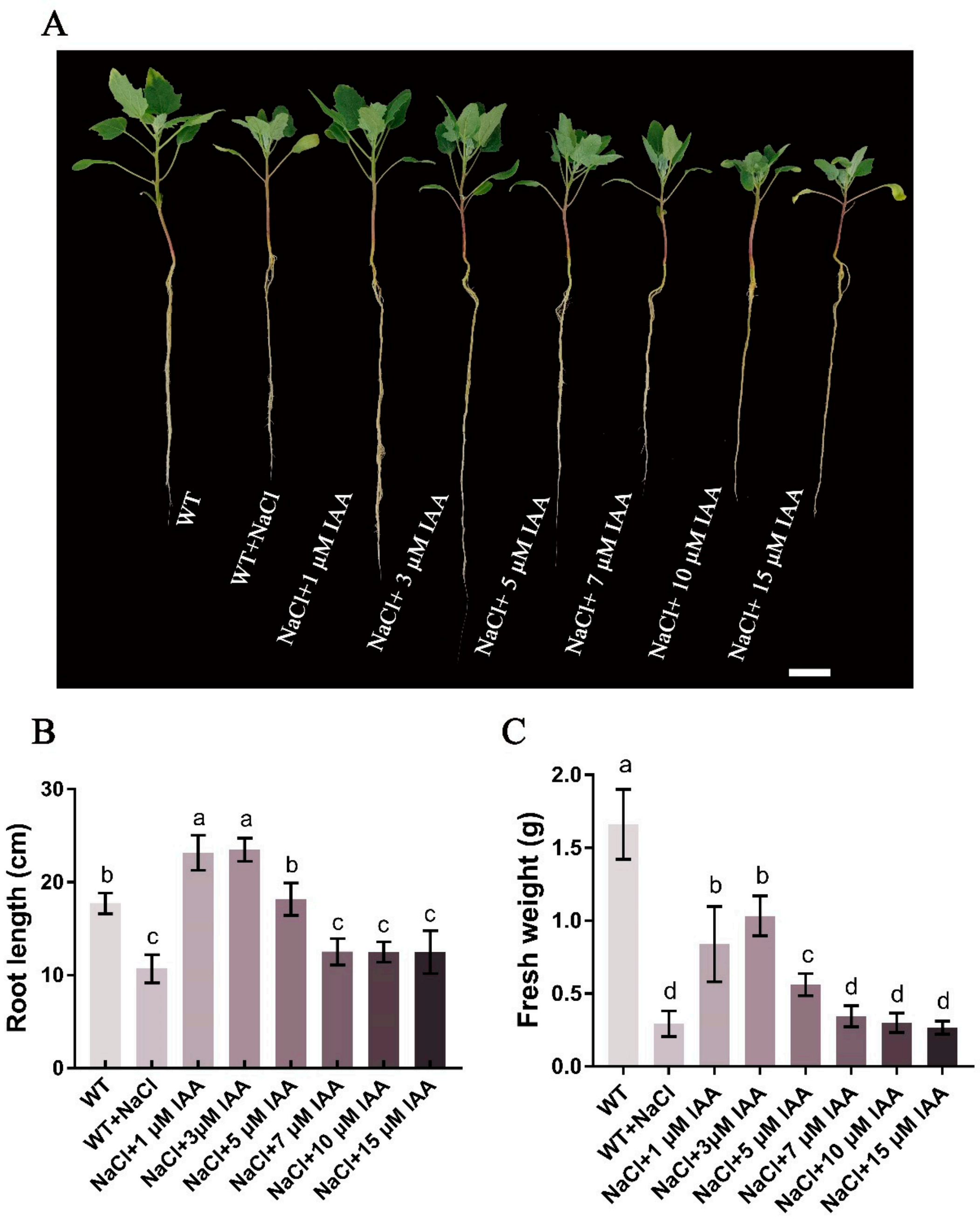
A
WT
WT+NaCl
NaCl+1 μM IAA
NaCl+ 3 μM IAA
NaCl+ 5 μM IAA
NaCl+ 7 μM IAA
NaCl+ 10 μM IAA
NaCl+ 15 μM IAA
B
Root length (cm)
0
10
20
30
b
c
a
a
b
c
c
c
WT
WT+NaCl
NaCl+1 μM IAA
NaCl+3μM IAA
NaCl+5 μM IAA
NaCl+7 μM IAA
NaCl+10 μM IAA
NaCl+15 μM IAA
C
Fresh weight (g)
0.0
0.5
1.0
1.5
2.0
a
d
b
b
c
d
d
d
WT
WT+NaCl
NaCl+1 μM IAA
NaCl+3μM IAA
NaCl+5 μM IAA
NaCl+7 μM IAA
NaCl+10 μM IAA
NaCl+15 μM IAA

Figure S2

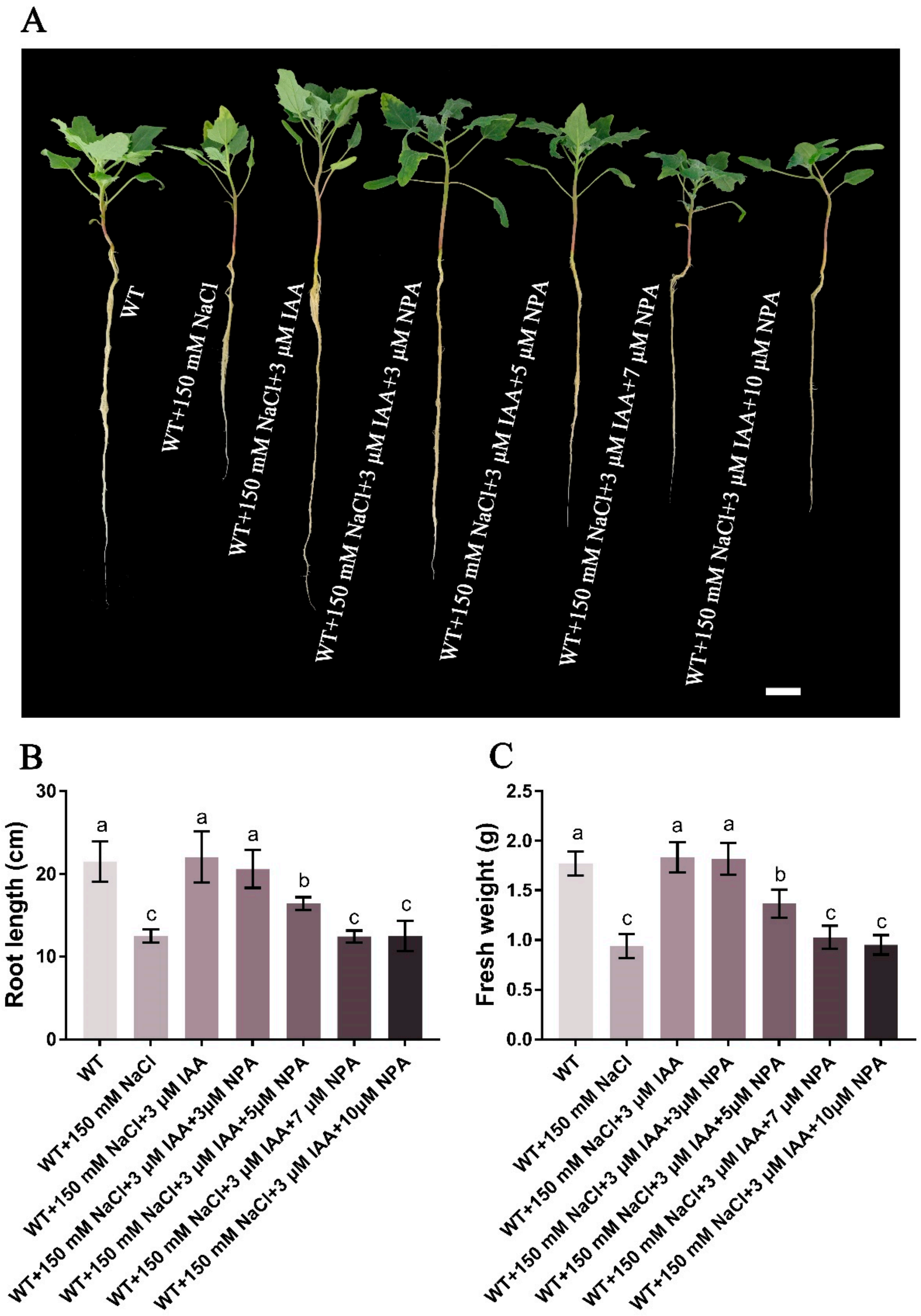
A
WT
WT+150 mM NaCl
WT+150 mM NaCl+3 μM IAA
WT+150 mM NaCl+3 μM IAA+3 μM NPA
WT+150 mM NaCl+3 μM IAA+5 μM NPA
WT+150 mM NaCl+3 μM IAA+7 μM NPA
WT+150 mM NaCl+3 μM IAA+10 μM NPA
B
Root length (cm)
0
10
20
30
a
c
a
a
b
c
c
WT
WT+150 mM NaCl
WT+150 mM NaCl+3 μM IAA
WT+150 mM NaCl+3 μM IAA+3μM NPA
WT+150 mM NaCl+3 μM IAA+5μM NPA
WT+150 mM NaCl+3 μM IAA+7 μM NPA
WT+150 mM NaCl+3 μM IAA+10μM NPA
C
Fresh weight (g)
0.0
0.5
1.0
1.5
2.0
2.5
a
c
a
a
b
c
c
WT
WT+150 mM NaCl
WT+150 mM NaCl+3 μM IAA
WT+150 mM NaCl+3 μM IAA+3μM NPA
WT+150 mM NaCl+3 μM IAA+5μM NPA
WT+150 mM NaCl+3 μM IAA+7 μM NPA
WT+150 mM NaCl+3 μM IAA+10μM NPA

Figure S3

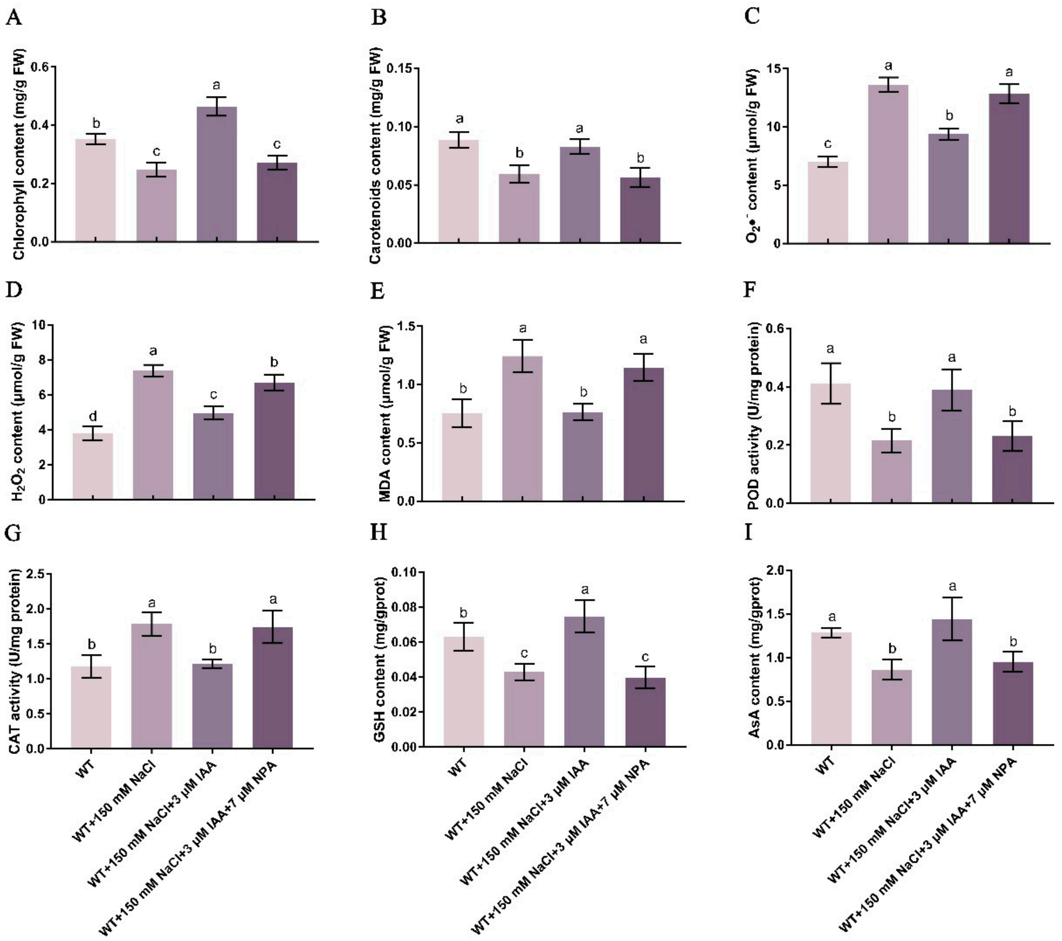

A
B
C
D
E
F
G
H
I
Chlorophyll content (mg/g FW)
Carotenoids content (mg/g FW)
$O_2^{\bullet-}$ content (μmol/g FW)
H_2O_2 content (μmol/g FW)
MDA content (μmol/g FW)
POD activity (U/mg protein)
CAT activity (U/mg protein)
GSH content (mg/gprot)
AsA content (mg/gprot)
WT
WT+150 mM NaCl
WT+150 mM NaCl+3 μM IAA
WT+150 mM NaCl+3 μM IAA+7 μM NPA

Figure S4

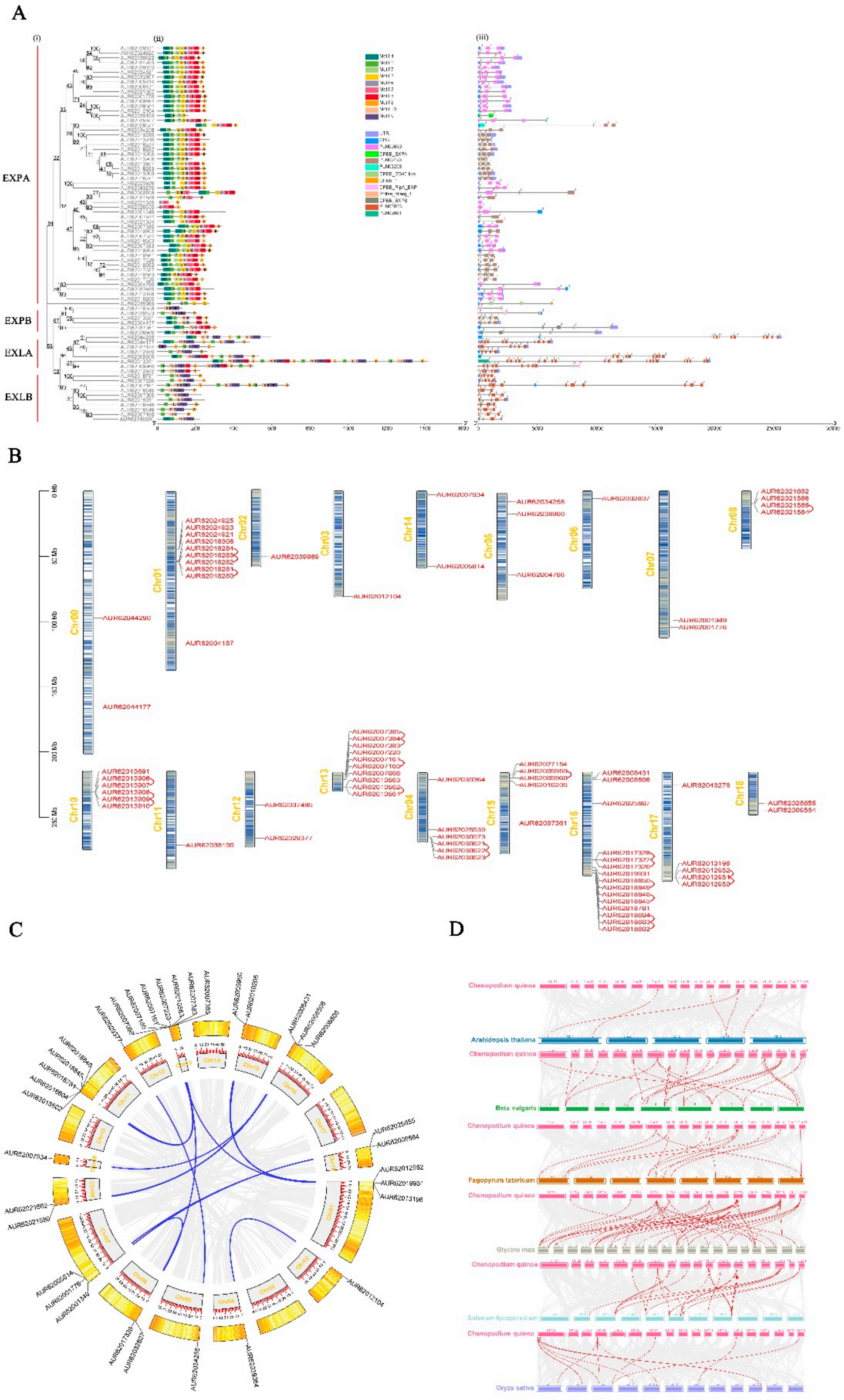
A
(i)
(ii)
(iii)
EXPA
EXPB
EXLA
EXLB
B
Chr00
Chr01
Chr02
Chr03
Chr04
Chr05
Chr06
Chr07
Chr08
Chr10
Chr11
Chr12
Chr13
Chr14
Chr15
Chr16
Chr17
Chr18
C
D
Chenopodium quinoa
Arabidopsis thaliana
Beta vulgaris
Fagopyrum tataricum
Glycine max
Solanum lycopersicum
Oryza sativa

Figure S5

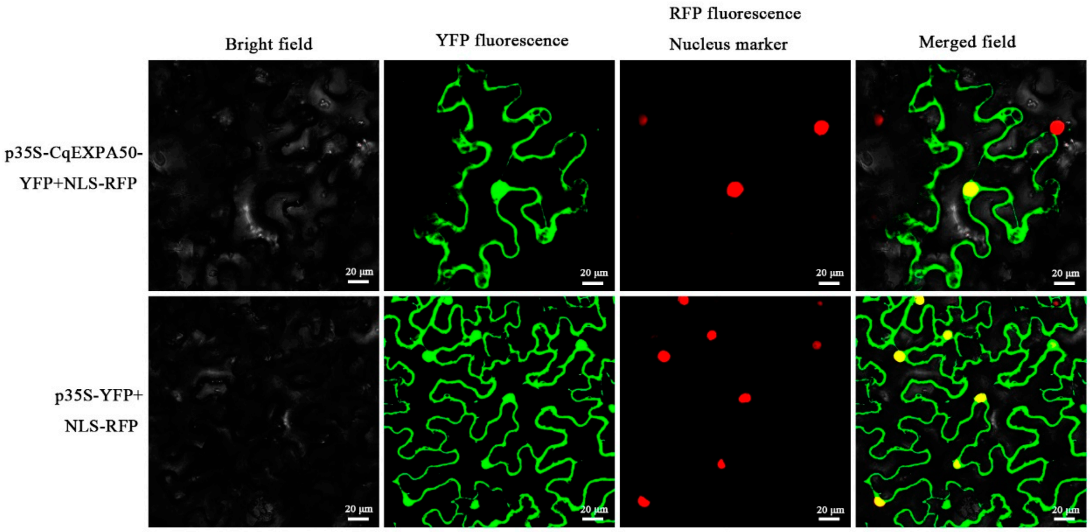

Bright field
YFP fluorescence
RFP fluorescence
Nucleus marker
Merged field
p35S-CqEXPA50-
YFP+NLS-RFP
p35S-YFP+
NLS-RFP
20 μm

Figure S6

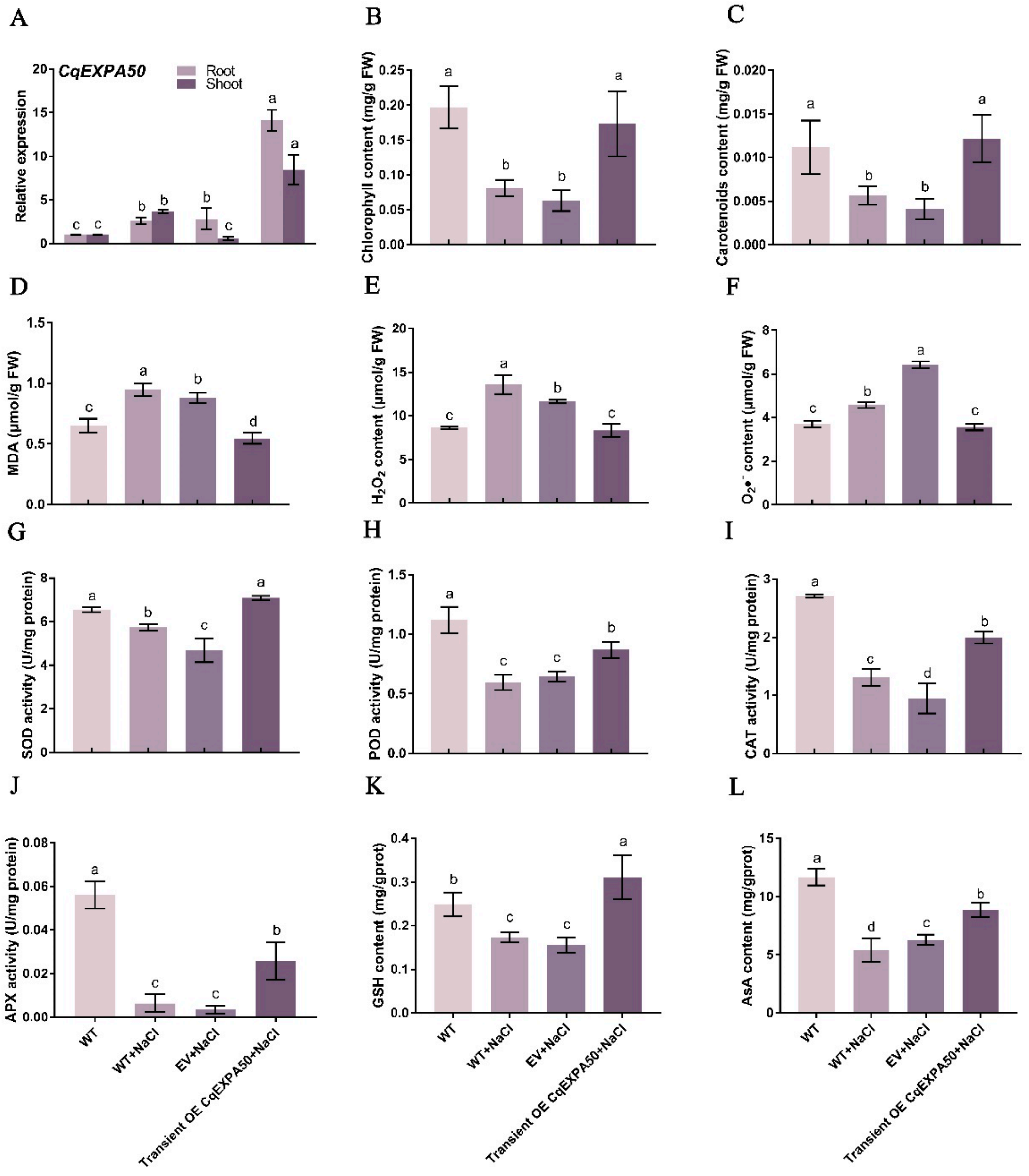
A
B
C
D
E
F
G
H
I
J
K
L
CqEXPA50
Root
Shoot
Relative expression
Chlorophyll content (mg/g FW)
Carotenoids content (mg/g FW)
MDA (μmol/g FW)
H_2O_2 content (μmol/g FW)
$O_2^{\bullet -}$ content (μmol/g FW)
SOD activity (U/mg protein)
POD activity (U/mg protein)
CAT activity (U/mg protein)
APX activity (U/mg protein)
GSH content (mg/gprot)
AsA content (mg/gprot)
WT
WT+NaCl
EV+NaCl
Transient OE CqEXPA50+NaCl

Figure S7

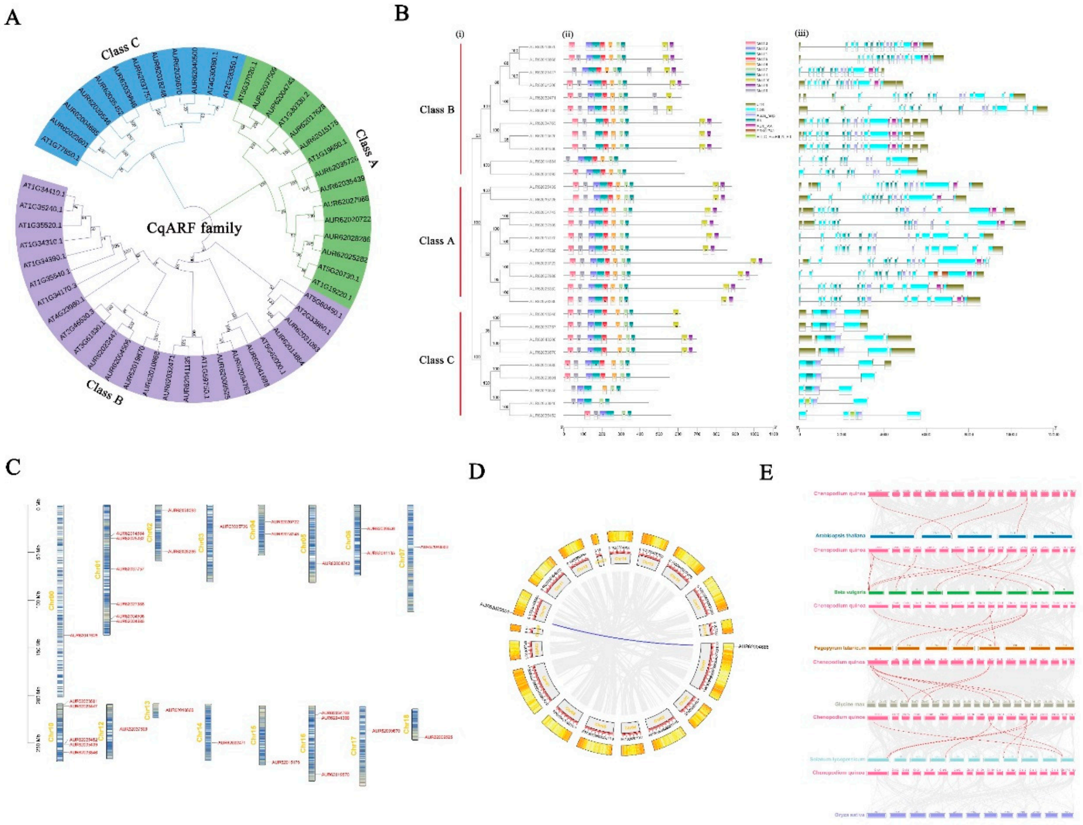

A
Class C
Class A
Class B
CqARF family
B
(i)
(ii)
(iii)
Class B
Class A
Class C
C
D
E

Figure S8

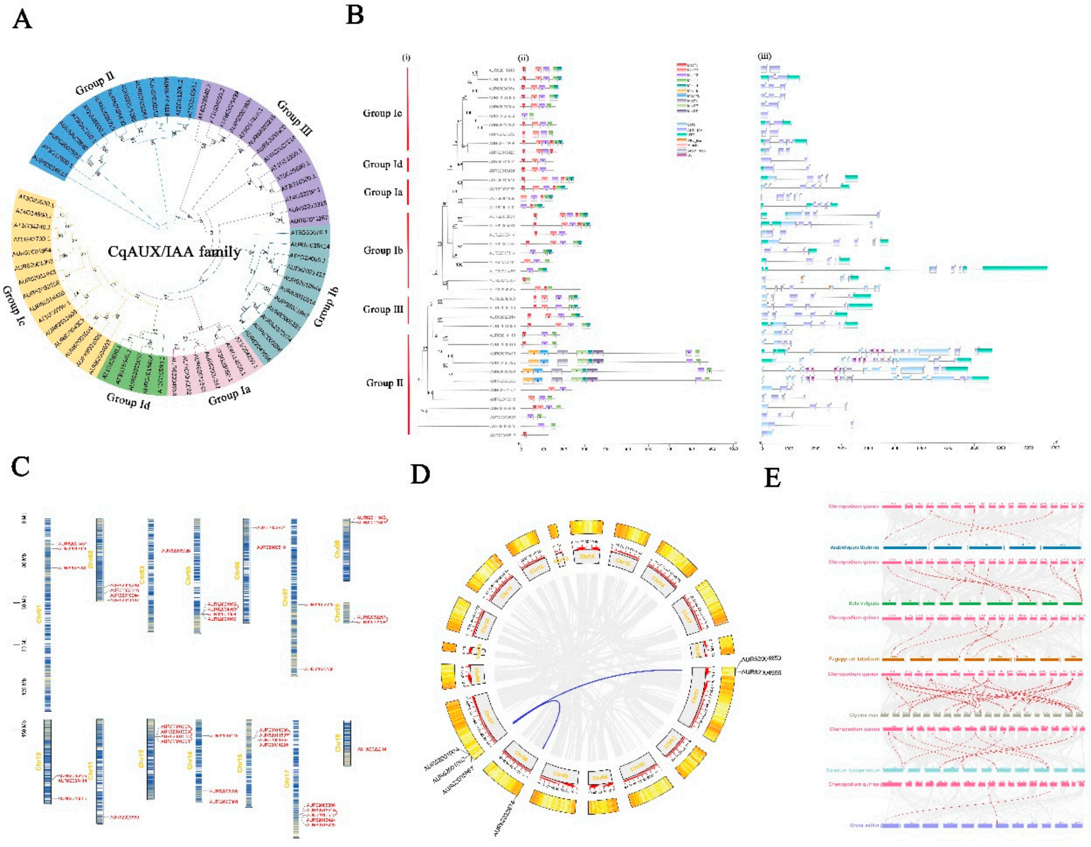
A
B
C
D
E
Group II
Group III
Group Ib
Group Ia
Group Id
Group Ic
CqAUX/IAA family
(i)
(ii)
(iii)
Group Ic
Group Id
Group Ia
Group Ib
Group III
Group II

Figure S9

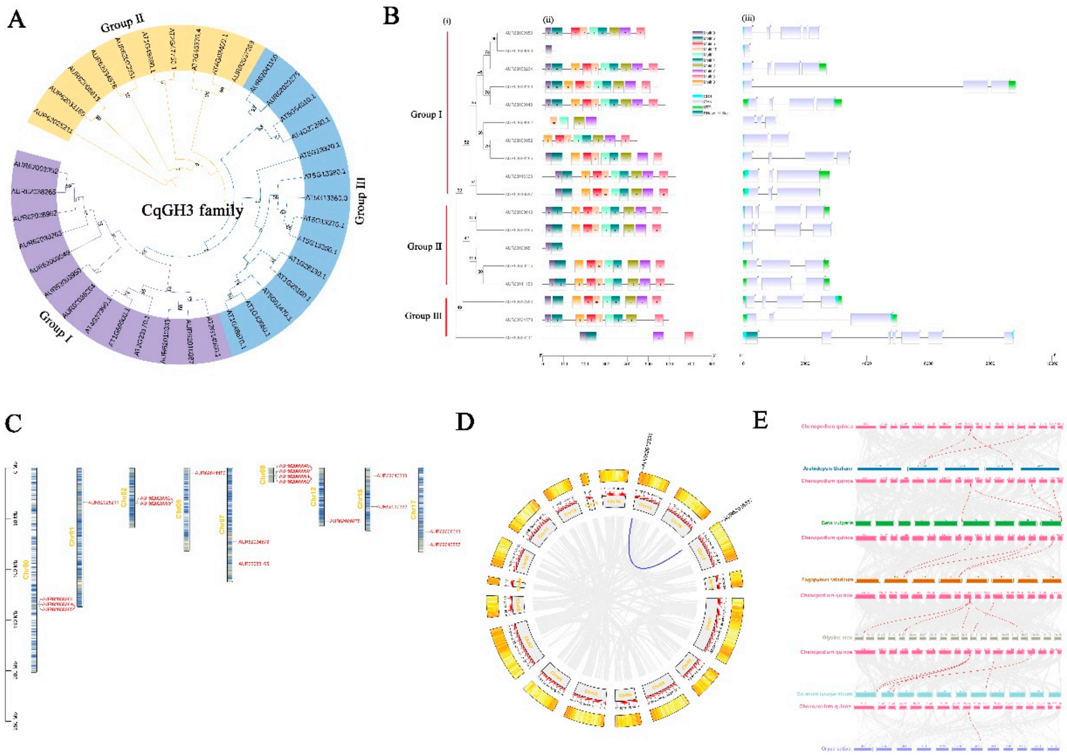
A
Group II
Group III
Group I
CqGH3 family
B
Group I
Group II
Group III
C
D
E

Figure S10

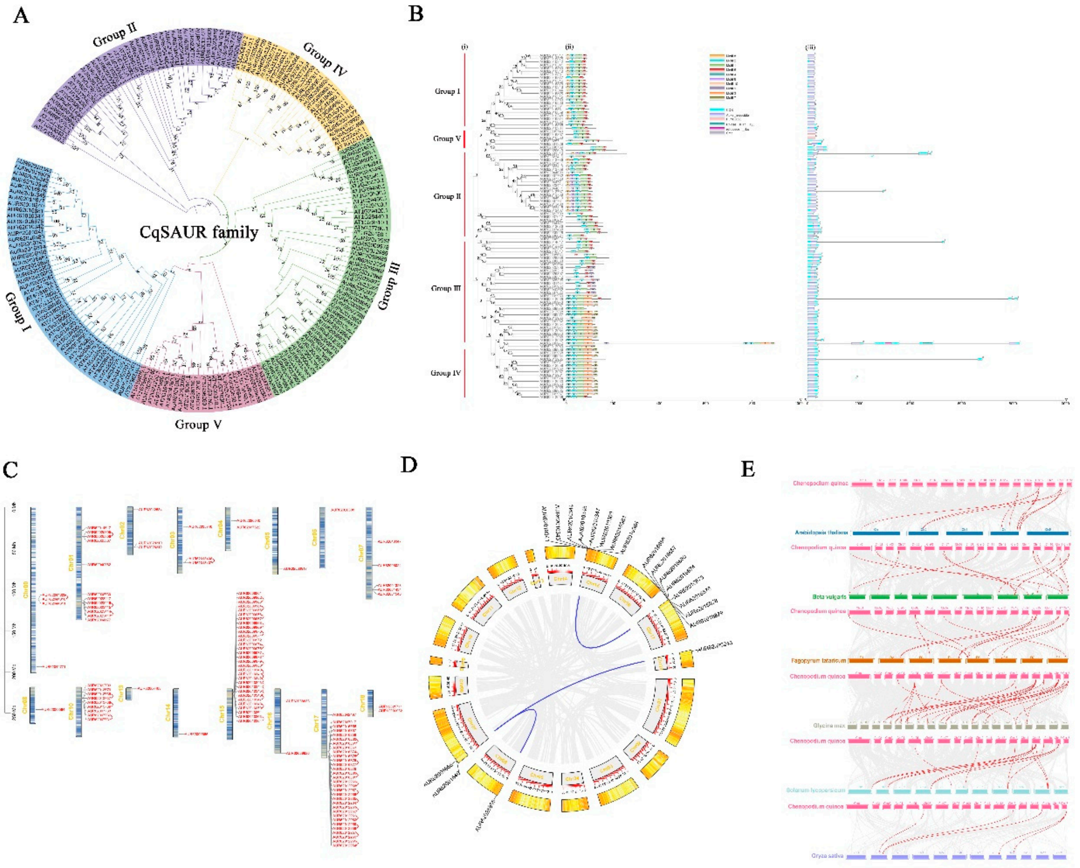
A
B
C
D
E
CqSAUR family
Group I
Group II
Group III
Group IV
Group V
Chenopodium quinoa
Arabidopsis thaliana
Beta vulgaris
Fagopyrum tataricum
Glycine max
Solanum lycopersicum
Oryza sativa

Figure S11

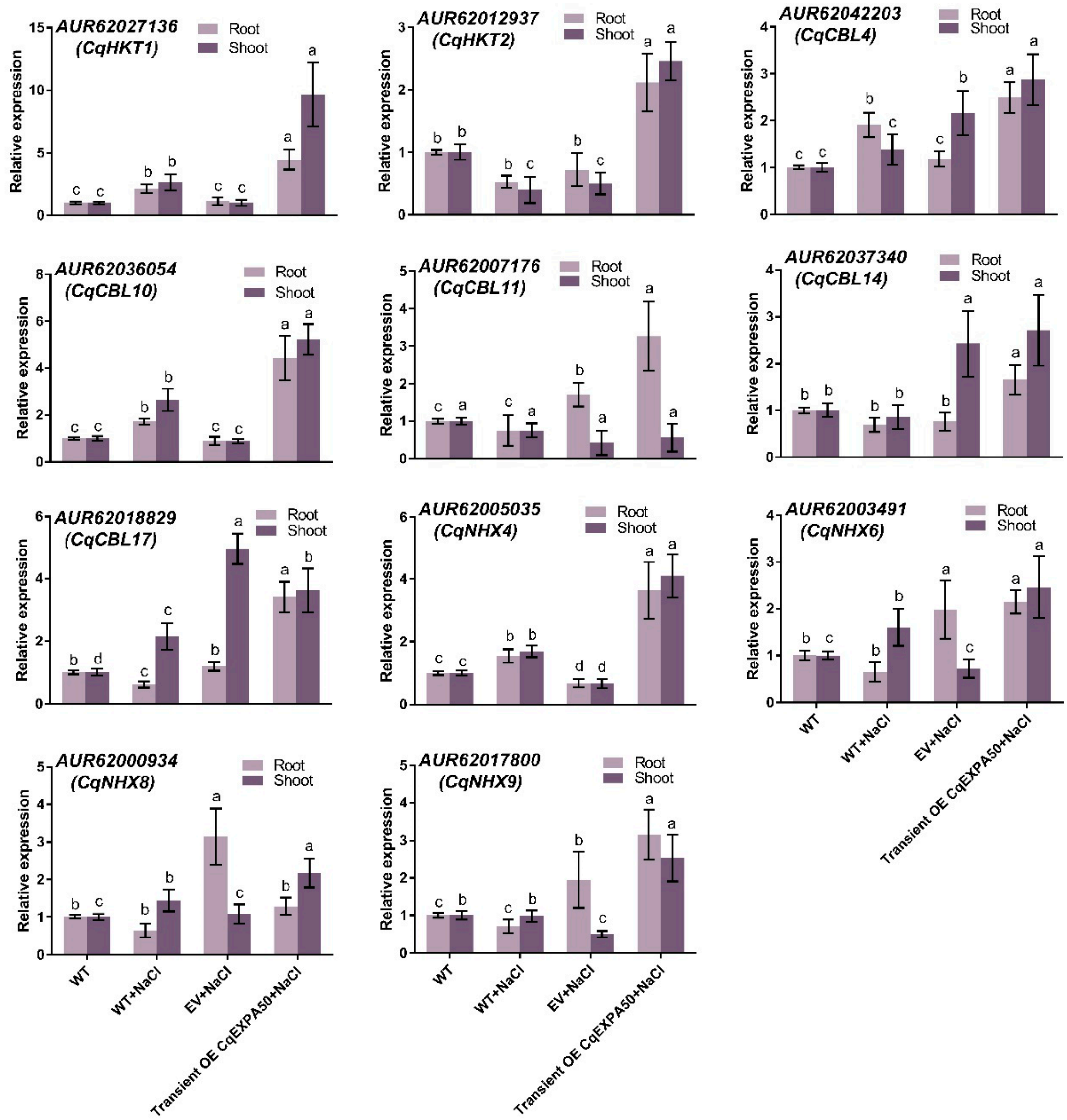

AUR62027136 (CqHKT1)
AUR62012937 (CqHKT2)
AUR62042203 (CqCBL4)
AUR62036054 (CqCBL10)
AUR62007176 (CqCBL11)
AUR62037340 (CqCBL14)
AUR62018829 (CqCBL17)
AUR62005035 (CqNHX4)
AUR62003491 (CqNHX6)
AUR62000934 (CqNHX8)
AUR62017800 (CqNHX9)
Root
Shoot
Relative expression
WT
WT+NaCl
EV+NaCl
Transient OE CqEXPA50+NaCl

Figure S12

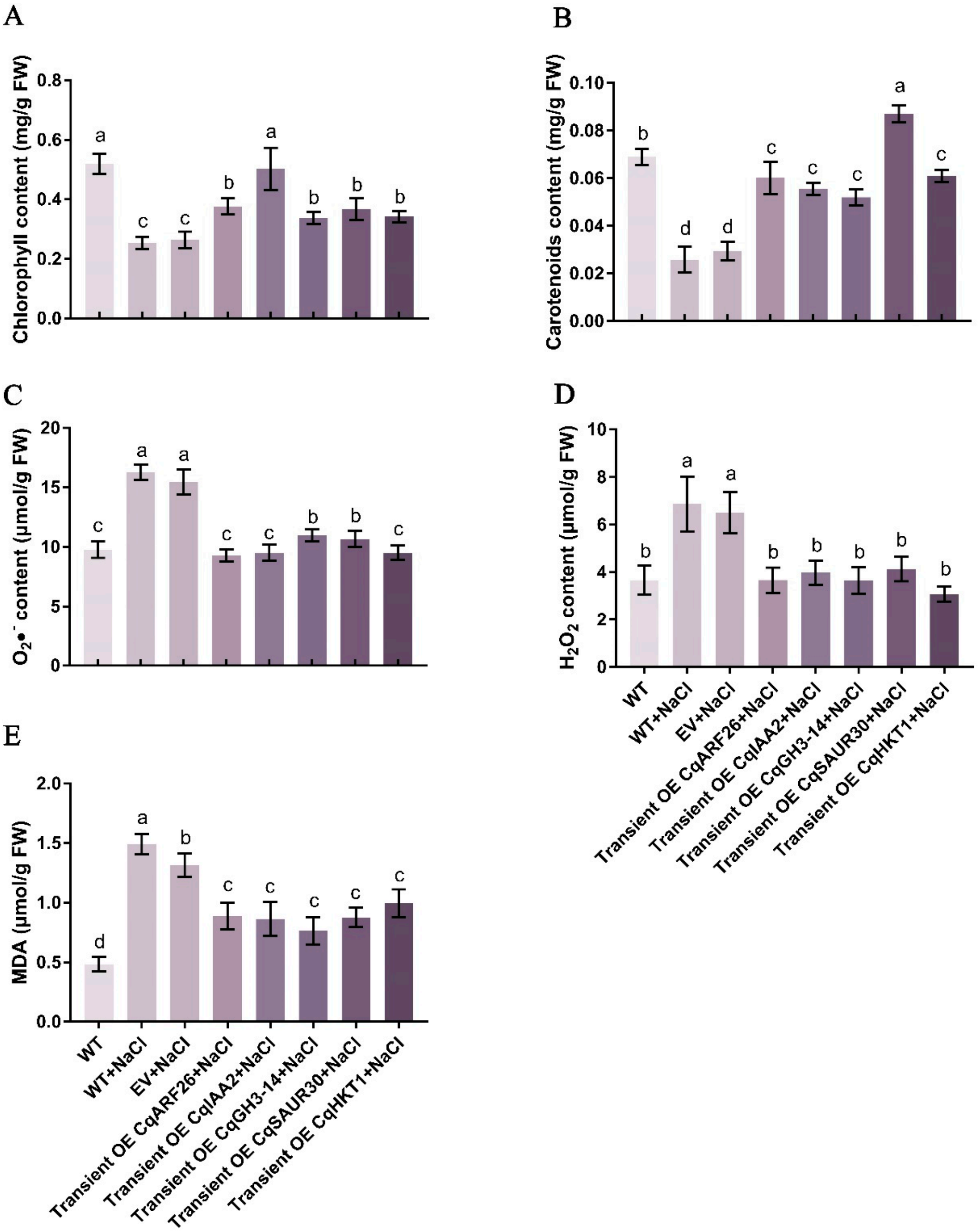
A
Chlorophyll content (mg/g FW)
B
Carotenoids content (mg/g FW)
C
$O_2\bullet^-$ content (μmol/g FW)
D
H_2O_2 content (μmol/g FW)
E
MDA (μmol/g FW)
WT
WT+NaCl
EV+NaCl
Transient OE CqARF26+NaCl
Transient OE CqIAA2+NaCl
Transient OE CqGH3-14+NaCl
Transient OE CqSAUR30+NaCl
Transient OE CqHKT1+NaCl

Figure S13

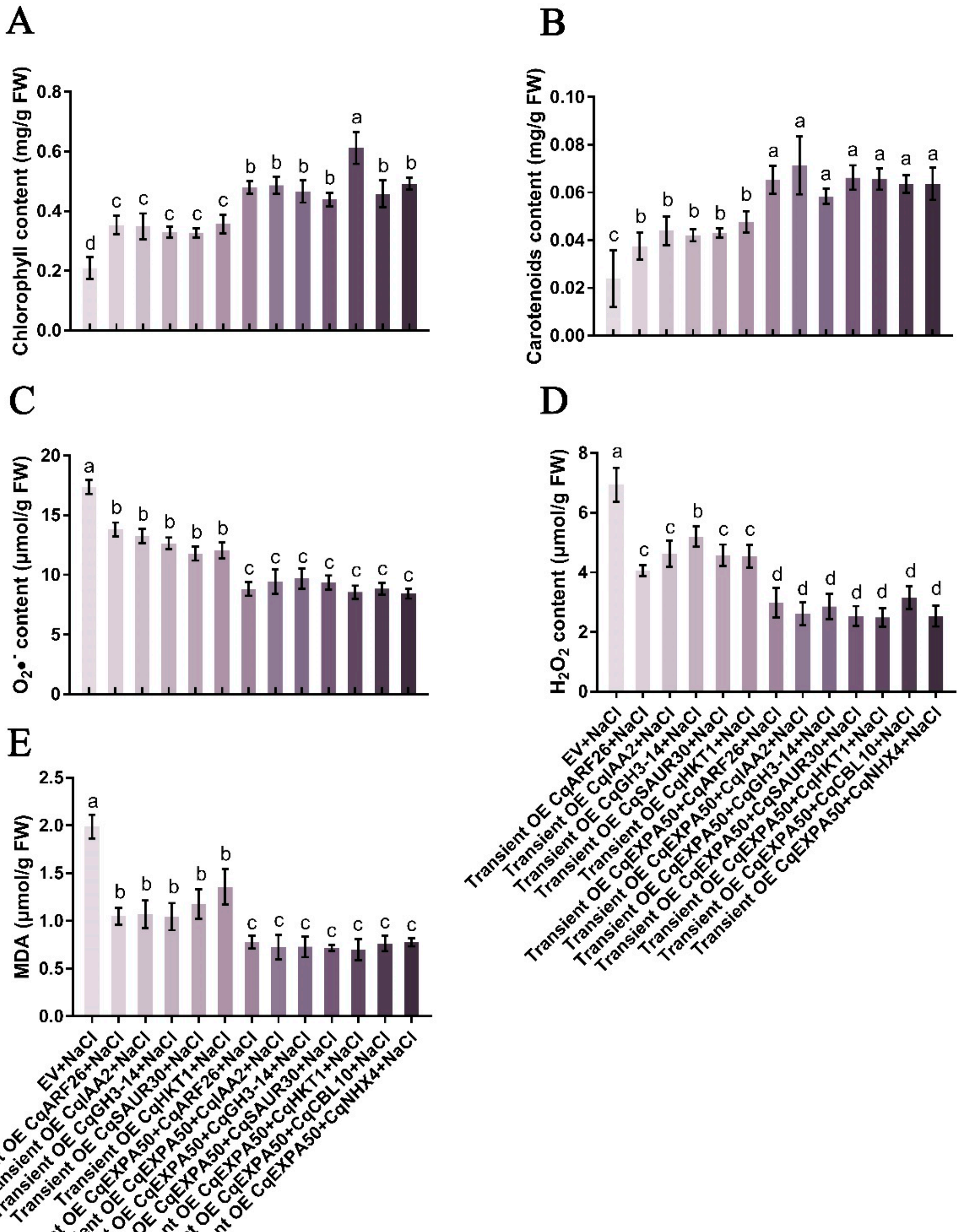

A
Chlorophyll content (mg/g FW)
B
Carotenoids content (mg/g FW)
C
O2•- content (µmol/g FW)
D
H2O2 content (µmol/g FW)
E
MDA (µmol/g FW)
EV+NaCl
Transient OE CqARF26+NaCl
Transient OE CqIAA2+NaCl
Transient OE CqGH3-14+NaCl
Transient OE CqSAUR30+NaCl
Transient OE CqHKT1+NaCl
Transient OE CqEXPA50+CqARF26+NaCl
Transient OE CqEXPA50+CqIAA2+NaCl
Transient OE CqEXPA50+CqGH3-14+NaCl
Transient OE CqEXPA50+CqSAUR30+NaCl
Transient OE CqEXPA50+CqHKT1+NaCl
Transient OE CqEXPA50+CqCBL10+NaCl
Transient OE CqEXPA50+CqNHX4+NaCl
